# Persulfide Dioxygenase From *Acidithiobacillus caldus*: Variable Roles of Cysteine Residues and Hydrogen Bond Networks of the Active Site

**DOI:** 10.3389/fmicb.2018.01610

**Published:** 2018-07-20

**Authors:** Patrick Rühl, Patrick Haas, Dominik Seipel, Jan Becker, Arnulf Kletzin

**Affiliations:** Department of Biology, Sulfur Biochemistry and Microbial Bioenergetics, Technische Universität Darmstadt, Darmstadt, Germany

**Keywords:** persulfide dioxygenase, sulfhydryl, sulfur, enzyme kinetics, differential scanning fluorimetry, ETHE1, glutathione persulfide, S-glutathionylation

## Abstract

Persulfide dioxygenases (PDOs) are abundant in Bacteria and also crucial for H_2_S detoxification in mitochondria. One of the two *pdo*-genes of the acidophilic bacterium *Acidithiobacillus caldus* was expressed in *Escherichia coli.* The protein (*Ac*PDO) had 0.77 ± 0.1 Fe/subunit and an average specific sulfite formation activity of 111.5 U/mg protein (*V*_max_) at 40°C and pH 7.5 with sulfur and GSH following Michaelis–Menten kinetics. *K*_M_ for GSH and *K*_cat_ were 0.5 mM and 181 s^−1^, respectively. Glutathione persulfide (GSSH) as substrate gave a sigmoidal curve with a *V*_max_ of 122.3 U/mg protein, a *K*_cat_ of 198 s^−1^ and a Hill coefficient of 2.3 ± 0.22 suggesting positive cooperativity. Gel permeation chromatography and non-denaturing gels showed mostly tetramers. The temperature optimum was 40–45°C, the melting point 63 ± 1.3°C in thermal unfolding experiments, whereas low activity was measurable up to 95°C. Site-directed mutagenesis showed that residues located in the predicted GSH/GSSH binding site and in the central hydrogen bond networks including the iron ligands are essential for activity. Among these, the R_139_A, D_141_A, and H_171_A variants were inactive concomitant to a decrease of their melting points by 3–8 K. Other variants were inactivated without significant melting point change. Two out of five cysteines are likewise essential, both of which lie presumably in close proximity at the surface of the protein (C_87_ and C_224_). MalPEG labeling experiments suggests that they form a disulfide bridge. The reducing agent Tris(2-carboxyethyl)phosphine was inhibitory besides *N*-ethylmaleimide and iodoacetamide suggesting an involvement of cysteines and the disulfide in catalysis and/or protein stabilization. Mass spectrometry revealed modification of C_87_, C_137_, and C_224_ by 305 mass units equivalent to GSH after incubation with GSSH and with GSH in case of the C_87_A and C_224_A variants. The results of this study suggest that disulfide formation between the two essential surface-exposed cysteines and Cys-S-glutathionylation serve as a protective mechanism against uncontrolled thiol oxidation and the associated loss of enzyme activity.

## Introduction

Persulfide dioxygenases (PDOs) catalyze the oxidation of glutathione persulfide (GSSH) and higher homologs (GSS_n_H; *n* > 1) with sulfite and reduced glutathione (GSH) as products (Tiranti et al., 2009; Kabil and Banerjee, 2012; Jung et al., 2016). Due to the high reactivity of reduced glutathione with elemental sulfur in the enzyme assay (Sluiter, 1930), PDOs were formerly classified as sulfur dioxygenases (SDO) because they produce sulfite from GSH-containing sulfur suspensions. SDO activities are known for a long time from chemolithotrophic sulfur-oxidizing bacteria of the genus (*Acidi-) Thiobacillus*, however, the protein was neither purified successfully nor the corresponding gene identified (Suzuki and Werkman, 1959; Suzuki, 1965a,b; Suzuki and Silver, 1966; Rohwerder and Sand, 2003). More recently, it was found that the human ethylmalonic encephalopathy protein 1 (hETHE1) has SDO/PDO activity. hETHE1 plays an important role in mitochondrial sulfide detoxification together with sulfide:quinone oxidoreductase and rhodanese (sulfane sulfur transferase; Hildebrandt and Grieshaber, 2008; Tiranti et al., 2009; Kabil and Banerjee, 2014). Mutations in the ETHE1 gene are responsible for the hereditary and fatal autosomal recessive disorder ethylmalonic encephalopathy (Tiranti et al., 2004, 2006, 2009; Di Meo et al., 2018), which is characterized by high levels of thiosulfate and C3–C5 compounds like ethylmalonic acid in urine and body fluids (Tiranti et al., 2006, 2009).

Triggered by the discovery of ETHE1 in human mitochondria, ETHE1-homologous proteins with PDO activity were recently identified from *Ac. caldus* (*Ac*PDO)*, Ac. ferrooxidans* and from several heterotrophic bacteria (Liu et al., 2014; Wang et al., 2014; Sattler et al., 2015), sometimes fused with a rhodanese domain (Shen et al., 2015; Motl et al., 2017). The bacterial PDOs seem to be responsible for the SDO activity observed earlier (Suzuki and Werkman, 1959; Suzuki, 1965a,b; Suzuki and Silver, 1966; Rohwerder and Sand, 2003).

X-ray structures of the human ETHE1 (PDB identifier 4CHL) and of ETHE1-like PDOs from *Arabidopsis thaliana* (2CGU) and several bacteria showed homodimeric or homotetrameric proteins with molecular masses of 25–30 kDa (or 40–45 kDa for the rhodanese fusion proteins; McCoy et al., 2006b; Pettinati et al., 2015; Sattler et al., 2015; Motl et al., 2017). The active sites each contain a mononuclear non-heme iron center coordinated by two histidines and one aspartate together with three water molecules. The resulting octahedral coordination sphere is known as 2-His-1-carboxylate facial triad and is typical for over 100 different oxygenases (for a recent review, see Kal and Que, 2017). The active site cavity comprises the GSH-binding residues, which position the substrate, so that the sulfur atom(s) bind to the iron displacing one or more of the water ligands as shown by the 3D structures of the PDOs from *Pseudomonas putida* (4YSL) and *Paraburkholderia phytofirmans* (*Pp*PDO; 5VE5; Sattler et al., 2015; Motl et al., 2017).

**FIGURE 1:**
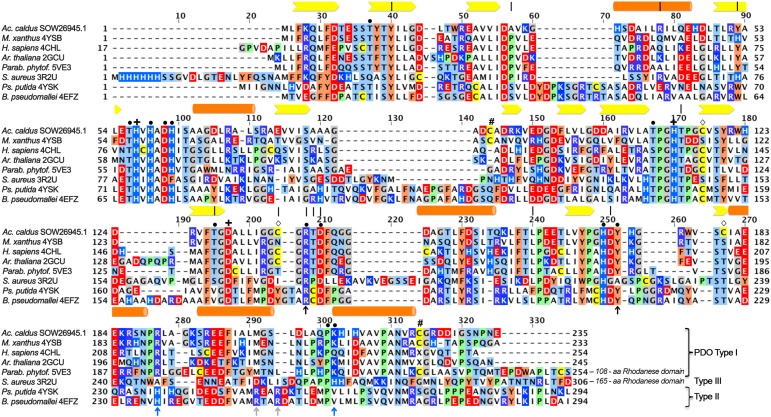
Multiple alignment of iron-containing persulfide dioxygenase (PDO) sequences. Sequences were downloaded from GenBank, aligned using the MAFFT server and manually corrected with respect to the 3D structures (GenBank/PDB accession in species names). Type I, Type II, and Type III enzymes were grouped according to Xia et al. (2017). Bars, alpha helices; arrows: beta sheets; **|**, residues correlated with ETHE according to Refs. (Tiranti et al., 2004, 2006; Jung et al., 2016); **+**, iron ligands; **#**, cysteine residues at the surface of the *Mx*PDO (Sattler et al., 2015); 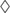, other cysteine residues of the *Ac*PDO; • secondary coordination sphere and hydrogen bonding network residues; black ↑, GSH-binding residues in all PDOs; blue ↑, GSH-binding residues in all PDOs; gray ↑, GSH-binding residues in Type II PDOs.

Persulfide dioxygenases are divided into three subfamilies, Type I–III (Liu et al., 2014; Xia et al., 2017). The human ETHE1 (4CHL) and the *Arabidopsis* PDO (2GCU) group in Type I together with many bacterial PDOs including two *Ac*PDOs, the *Pp*PDO and the *Myxococcus xanthus* enzyme (*Mx*PDO; 4YSB, **Figure 1**; Pettinati et al., 2015; Sattler et al., 2015). The Type II PDO from *Ps. putida* is larger than Type I enzymes due to additional loops. The Type III enzyme from *Staphylococcus aureus* has a C-terminal rhodanese domain similar to the *Pp*PDO but a different substrate specificity: bacillithiol and coenzyme A persulfides exceed GSSH and cysteine persulfide 10- to 20-fold in terms of their respective specificity constants (Shen et al., 2015). They all belong to the metallo-β-lactamase (MBL) protein superfamily together with glyoxalase II (PFAM database PF00753).

Several of the amino acid residues, whose mutations cause ETHE in humans, are conserved among the PDOs. They are either part of the GSH binding pocket or the extended hydrogen bond network around the iron site. Some of the latter residues coordinate another metal ion in MBL and glyoxalase II, most of which contain dinuclear zinc in the active sites (Cameron et al., 1999; Sattler et al., 2015; for a recent review, see Meini et al., 2015). Arg_163_ is one prominent example among these residues in hETHE1: mutations here alter protein stability and reduction potential of the iron (**Figure 1**; Tiranti et al., 2006; Henriques et al., 2014). In spite of the obvious importance, no comprehensive mutagenesis study has been published so far addressing the roles of individual residues in the H-bond network.

Lin et al. (2016) published the first theoretical study recently on the reaction mechanism of the human PDO applying quantum mechanics/molecular mechanics coupled to molecular dynamics calculations. They concluded that “*… the ground state of the iron(II)-superoxo reactant is quintet, which can be described as GSS^+⋅^-Fe(II)-O_2_^⋅^, and the most feasible reaction channel was found to start from the cleavage of dioxygen and a concerted attack of distal oxygen on the sulfur atom of the substrate, forming the metal-bound activated oxygen and a sulfite intermediate.*” They also predicted that hETHE1-His_81_, located in the vicinity of the iron site and part of a conserved amino acid motif around the iron ligands (**Figure 1**; Sattler et al., 2015), plays an important role in binding of the persulfide moiety of GSSH. The study did not include a possible participation of one or more of the cysteines or the role of the hydrogen bond network around the active site.

Some of the cysteine residues in PDOs seem to be important for catalysis and there is evidence that C_247_ of the hETHE1 is essential not only for enzyme activity but also for an inferred polysulfidation of other cysteine residues in the protein (Jung et al., 2016). C_247_ is present in the oxidation state of sulfinic acid (Cys-SO_2_^−^) in the crystal structures of the hETHE1 and the *Arabidopsis* enzyme but not in the bacterial proteins (McCoy et al., 2006b; Pettinati et al., 2015; Sattler et al., 2015; Motl et al., 2017). Inhibition studies of the *Ac*PDO with thiol-binding reagents also indicated the importance of the cysteine residues (Wang et al., 2014; Jung et al., 2016). Interestingly, the cysteine residues are not conserved among the various PDOs and their numbers vary (**Figure 1**) so that the function of the cysteine(s) is unresolved.

Here we describe biochemical properties of the bacterial *Ac*PDO including enzyme kinetics, inhibition properties, and melting points. In two publications, the temperature and pH dependences and the kinetic constants of two different PDOs from *Ac. caldus* MTH-04 had been reported (GenBank locus tags A5904_0790 and A5904_0421; Wang et al., 2014; Wu et al., 2017). We repeated these measurements with the PDO from *Ac. caldus* strain C-SH12 (Hallberg and Lindström, 1994; homologous to A5904_0790) and a standardized and optimized assay resulting in much higher specific activities. We also present an extensive mutagenesis study based upon a homology model with the similar *Mx*PDO and *Pp*PDO as templates and including the cysteines and the residues of the hydrogen bond network in order to define amino acids essential for activity. We also show that different cysteine modifications can be identified (glutathionylation and/or disulfide bridge formation). The results are discussed with a focus on the contribution of individual residues for PDO activity in the light of the structural model.

## Materials and Methods

### Vector Construction and Heterologous Gene Expression

*Acidithiobacillus caldus* C-SH12 (DSM 9466; Hallberg and Lindström, 1994) was obtained from the Deutsche Sammlung für Mikroorganismen und Zellkulturen (Braunschweig, Germany). Cells were grown at 37°C in medium No 670 with elemental sulfur as the sole electron donor with atmospheric CO_2_^1^. Genomic DNA was extracted from a culture volume of 20 ml using the GenElute bacterial genomic DNA kit (Sigma-Aldrich, Munich, Germany). The *pdo* gene (EMBL accession no: PRJEB24175) was PCR-amplified with the primers Acical_ETHE1_fwd and Acical_ETHE1_rev (Supplementary Table S1). The PCR product was purified using the GenElute PCR Clean-Up Kit (Sigma-Aldrich) and subsequently digested with *Xba*I at the 5′ end of the gene, whereas the 3′ end was not restricted [all restriction and modification enzymes by New England Biolabs (NEB), Frankfurt am Main, Germany]. The vector pASK75 (Skerra, 1994) was linearized with *Xba*I, *Xho*I, *Eco*RI, and *Afe*I simultaneously, dephosphorylated with Antarctic phosphatase after the manufacturer’s recommendations. The enzymes were heat-inactivated for 20 min at 80°C. The PCR product was phosphorylated using T4 polynucleotide kinase in 1× T4 Ligation buffer followed by heat inactivation of the kinase. One hundred nanograms of the pre-digested Vector DNA and a threefold molar excess of the PCR product were added to the ligation reaction in a final volume of 20 μl containing 2 μl of 10× ligation buffer and 1 μl of T4 DNA ligase. The reaction was incubated for 4 h at room temperature. *Escherichia coli* Top 10F’ cells (Life Technologies, Darmstadt, Germany) were transformed with the ligation mixture and the resulting plasmid pASK_*Ac*PDO was isolated from positive transformants and sequenced for confirmation. The plasmid was finally introduced into *E. coli* BL21(DE3) CodonPlus RIL cells (Agilent, Böblingen, Germany) for gene expression.

For protein production, 500 ml cultures were grown in LB medium at 37°C in notched Erlenmeyer flasks with vigorous shaking after inoculation from a 20 ml overnight culture to an approximate OD_600_ of 0.05. Gene expression was induced at an OD_600_ between 0.6 and 0.8 by addition of 200 μg/l anhydrotetracycline (Iba, Göttingen, Germany) from a 0.2% [w/v] stock solution in dimethylformamide. Ferric citrate was added to 100 μM at the time of induction to ensure sufficient iron incorporation. The Fe(III)citrate stock solution each contained 100 mM citric acid and FeCl_3_. The cultures were incubated for 20 h after induction with vigorous shaking (180 rpm).

### Modeling and Site-Directed Mutagenesis

3D models of the *Ac*PDO were built using the Phyre^2^ and the I-Tasser servers (Kelley et al., 2015; Yang et al., 2015). The Phyre^2^ server was also used to build 1:1 threading models of the *Ac*PDO with the *Mx*PDO and *Pp*PDO (Sattler et al., 2015) as templates. The figures were prepared in PyMol (DeLano, 2002).

Mutants of several codons were constructed using inverse PCR amplification of the pASK_*Ac*PDO plasmid with the appropriate back-to-back mutagenesis primers containing the desired mutation (Supplementary Table S1; Hemsley et al., 1989) and an additional silent mutation for the introduction or deletion of a restriction enzyme recognition site in order to differentiate mutagenized plasmids from wild type by restriction digestion. PC reactions were performed in 50 μl reaction volume containing 2.5 U Q5 DNA polymerase with the buffer supplied by the manufacturer (NEB), 25 pmol of each primer (Sigma-Aldrich; Munich, Germany), 250 μM dNTPs (Carl Roth; Karlsruhe, Germany), and ≈1 ng of the wild type plasmid. The PCR products were digested for 2 h with 10 U of *Dpn*I (Weiner et al., 1994), subsequently purified via the GenElute PCR Clean-Up Kit (Sigma-Aldrich; Munich, Germany) and eluted from the columns with 17 μl of elution buffer. 2 μl 10× DNA Ligase buffer, 10 U T4 polynucleotide kinase and 400 U T4 DNA ligase were added. The reaction mixture was incubated for 2 h at 37°C followed by heat inactivation (10 min at 80°C) and transformation of *E. coli* TOP 10F’ (Invitrogen/Fisher Scientific; Schwerte, Germany) cells with 10 μl of the total reaction mixture. The resulting constructs were analyzed by restriction digestion and sequencing (Seqlab, Göttingen, Germany or Eurofins Genomics, Ebersberg, Germany).

### Protein Purification

The cell pellet obtained by centrifugation was washed once in ≈50 vol (v/w) of 100 mM Tris/HCl buffer pH 8 with 150 mM NaCl (buffer W; Iba, Göttingen, Germany) and afterwards resuspended in 5 vol of the same buffer. Cells were disrupted by sonication for 10 min [Branson Sonifier 250, level 5, microtip (5 mm) and 100% duty cycle]. After a first centrifugation step (10,000 × *g* for 10 min), the soluble protein-containing supernatant was centrifuged in an ultracentrifuge (100,000 × *g* for 45 min). The supernatant was passed through a 0.22 μm syringe filter and applied to a 1 ml-Strep-Tactin XT Superflow column (Iba) connected to a peristaltic pump with a flow rate of ≈0.5 ml/min followed by 5 column volumes (CV) of buffer W at a flow rate of 1 ml/min. The protein was eluted from the column with a biotin-containing elution buffer (BXT) according to the manufacturer’s instructions (Iba). The column was regenerated with 2 CV of 10 mM NaOH followed by addition of 8 CV buffer W, both with a flow rate of 1 ml/min. Alternatively, standard 1 ml gravity-flow Streptactin columns were used with 2.5 mM desthiobiotin in buffer W and regeneration of the column with a 1 mM HABA in buffer W and 100 mM Tris base solution in water (no pH adjustment) followed by rinsing of the column with 5 CV of buffer W according to the manufacturer’s recommendations (Iba).

### Biochemical Procedures and Gel Shift Assay

The protein concentration was determined with the Bradford method (Bradford, 1976). Iron quantification was performed with purified protein preparations using the 2,4,6-tripyridyl-1,3,5-triazine method (TPTZ; Fischer and Price, 1964). Gel permeation chromatography was performed using an Äkta system with a 10/300 Superose 6 column (GE Healthcare, Freiburg, Germany) and cytochrome *c*, conalbumin, aldolase and catalase as standards (all Sigma-Aldrich). Denaturing sodium dodecyl sulfate gel electrophoresis (SDS-PAGE) was performed using 10% acrylamide Tris-tricine gels (Schägger and von Jagow, 1987). Native polyacrylamide gel electrophoresis was performed using SERVAGel N 4-16, vertical native gels (Serva, Heidelberg, Germany). For Western analysis, purified enzyme was separated by SDS-PAGE as described above. Subsequently, the proteins were transferred to a PVDF membrane (Roti-Fluoro PVDF, Roth) for 1.5 h at 0.8 mA/cm^2^ (Bio-Rad Laboratories). The membrane was incubated overnight at room temperature in blocking buffer (5% BSA in PBS buffer). The *Ac*PDO with a C-terminal Strep-tag was detected by the incubation with a *Strep*MAP-Classic HRP-conjugated antibody (IBA, Göttingen, Germany) and chloronaphthol staining of the membrane according to the manufacturer’s instructions. The chromogenic reaction was stopped by washing several times with distilled H_2_O.

Cysteine modifications were analyzed using a gel shift assay after derivatization of the protein with MalPEG (methoxypolyethylene glycol maleimide; MW 5,000; Sigma-Aldrich). For this purpose, 0.5 mg/ml PDO in buffer W (pH 7.2) were incubated with 5 mM NEM for 30 min at 30°C and subsequently derivatized with 2.5 mM MalPEG under identical conditions. For derivatization under reducing conditions, 5 mM DTT was added after the NEM incubation step. After 30 min of incubation, DTT and excess NEM were removed using spin columns (Roti-Spin MINI-3, 3 MWCO; Roth) and three washing steps with buffer W (pH 7.2) and the protein was derivatized with MalPEG as described above. The reaction was finally stopped by addition of 0.3 vol. of non-reducing SDS loading buffer (60 mM Tris/HCl 6.8, 15% glycerol, 9% SDS, 0.075% bromophenol blue).

### Thermal Unfolding

Differential scanning fluorimetry (DSF) displays the increase of tryptophane/tyrosine fluorescence upon temperature-dependent unfolding, traced in glass capillaries with a Prometheus NT.48 nanoDSF instrument (NanoTemper Technologies; Munich, Germany). The fluorescence ratio of 330/350 nm was recorded continuously during the experiment at a heating rate of 1°C/min. The melting points were calculated from the first derivative of the resulting melting curve. The protein concentration was 1 mg/ml in 100 mM Tris-HCl buffer (pH 8) containing 150 mM NaCl.

### PDO Activity Assays

For PDO activity assays, the enzyme reaction buffer [70 mM Tris/HCl pH 7.5 unless specified otherwise; 0.1% Tween20, 2% (wt/vol) sulfur flower] was sonicated for 5 min for sulfur dispersal (Branson, level 10, macrotip and 100% duty cycle). Aliquots of 2 ml were transferred to reaction tubes and 1 mM reduced glutathione was added from a 50 mM stock solution (range: 0.2–7.5 mM). The enzyme reaction buffer was used without sulfur flower if GSSH was the substrate. The reaction mixture was preheated for 5 min to the assay temperature of 40°C in a thermomixer with vigorous shaking (800 rpm). Usually, final enzyme concentrations of 1–2.5 μg/ml were used. 250 μl of the reaction mixture were transferred to 50 μl of a fuchsine solution in a 1.5 ml reaction vial immediately after enzyme addition to stop the reaction and provide a starting point for enzyme kinetics [0.04% fuchsine (wt/vol) in 12.5% sulfuric acid; Kletzin, 1989]. Similarly, additional 250 μl aliquots were transferred to identical vials in 10 s intervals for a period of 50 s. Immediately afterwards, 200 μl of distilled water were added to each of the vials and the mixtures were incubated for 5 min. Subsequently, 5 μl of 37% formalin were added to each tube, mixed and centrifuged for 1 min at 13,000 × g to sediment elemental sulfur. The supernatant was transferred to a half-micro cuvette and the absorbance was read at 570 nm after 60 min incubation time against a reagent blank. Thiosulfate production was determined as described previously (Kletzin, 1989; Urich et al., 2004). The colorimetrically determined amounts of sulfite and thiosulfate were summed up at each time point to give the final amount of products. The specific activities were calculated from the linear increase of the reaction products. One Unit (U) of enzyme activity was defined as 1 μmol of sulfite plus thiosulfate formed per minute.

The protein concentration was varied between 1 and 100 μg/ml for recording of the pH and temperature dependencies and for the measurement of mutagenized protein versions, depending on the expected activities under the given assay conditions. The optimal temperature of the *Ac*PDO activity was determined at pH 7.5, the pH profile was recorded at 45°C in 70 mM Tris buffer, using the same assay and pH values adjusted with HCl.

Reconstitution of the variants of the putative histidine ligands of the iron atom (H_57_A, H_57_G, H_113_A, and H_113_G) was attempted with addition of 2.5–30 mM imidazole and 50 μM Fe(III)Cl_3_ to the enzyme reaction buffer and activity assays. The specific activities of the proteins were determined against a reagent blank as described above. Alternatively, 100 μg of purified PDO (H_57_G) was incubated at 4°C in a total volume of 1 ml with 50 μM Fe(III)Cl_3_, 50 μM DTT and 2.5 mM imidazole for 18–24 h followed by dialysis against enzyme assay buffer free of iron and imidazole followed by determination of the specific activities.

### GSSH Synthesis and Quantification

Glutathione persulfide production was performed by two different methods, the first one as described previously with minor modifications (Visser et al., 1997; Liu et al., 2014). About 20 mM of sulfur flower were dissolved in acetone mixed with an equal volume of 20 mM GSH in potassium phosphate buffer pH 7.5. After incubation for at least 5 min at room temperature, the acetone was evaporated by centrifugation in a Speed-Vac vacuum centrifuge for 15 min at 30°C, before quantification of GSSH using cold cyanolysis (Wood et al., 1987). One hundred microliters of GSSH-containing reaction mixture was added to 80 μl of 1 M NaOH, 670 μl distilled water and 100 μl of 0.5 M potassium cyanide. The mixture was incubated for 45 min followed by addition of 20 μl of 37% formalin and 200 μl of Goldstein’s reagent (Goldstein, 1950). The absorbance was determined at 460 nm. A standard curve was prepared with potassium thiocyanate. The average GSSH concentration of the resulting solution was 2 mM. A second method for GSSH generation consisted of adding GSH at a final concentration of 1 mM to the sulfur-containing reaction buffer (pH 7.5) and heating of the mixture for 10 min at 80°C. Elemental sulfur was sedimented by centrifugation. Samples of the resulting GSSH preparations were derivatized with 48 mM Monobromobimane (Rethmeier et al., 1997) and subjected to mass spectrometry (MS).

### Mass Spectrometry

Mass spectrometry of GSSH and the *Ac*PDO holoprotein was performed at the MS unit of the Dept. of Chemistry (Technische Universität Darmstadt)^2^. For GSSH analysis, the mass spectrometer (Impact II, quadrupole-time-of-flight, Bruker Daltonik, Bremen, Germany) was equipped with an electrospray ion source operated in positive ion mode at 180°C source temperature. The capillary voltage was set to 3 kV with a nebulization pressure of 0.4 bar and a nitrogen stream at a flow rate of 4.0 l/min.

Holoprotein analysis was performed with electron spray ionization and MALDI-TOF MS directly from solution. 20 μg of freshly prepared *Ac*PDO was incubated for 1 min in a final volume of 100 μl enzyme reaction buffer (1) as prepared, (2) with 1 mM GSH or (3) 1 mM GSSH prepared by the first method described above (GSSH synthesis and quantification). Prior to analysis, samples were applied to a C4 HPLC column with a solvent mixture of H_2_O containing 0.01% TFA for 3 min followed by a gradient to 100% acetonitrile containing 0.01% TFA. The mass spectrometer (Impact II, Bruker Daltonik) was equipped with an electrospray ion source operated in positive ion mode. The selected mass range was between 800 and 5,000 m/z. Nitrogen was used as carrier gas and the temperature was set to 220°C with a nebulization pressure of 1.8 bar, a flow rate of 8 l/min and a capillary voltage of 4.5 kV. MALDI-TOF MS of the holoenzyme was carried out with an Autoflex speed TOF/TOF spectrometer (Bruker, Daltonik) with a 2,5-Dihydroxyacetophenone (DHAP) matrix.

MALDI-TOF MS of tryptic peptide fragments was performed at the MS unit of the University of Hohenheim (Stuttgart, Germany)^3^ after sample preparation following two different protocols. For the first approach, 20 μg each of the *Ac*PDO samples were incubated with 5 mM iodoacetamide (IAA; Sigma-Aldrich, Munich, Germany) for 45 min in the dark followed by separation by SDS-PAGE gel with and without DTT as a reductant in the SDS sample buffer. For control, as-isolated protein was separated and measured without IAA treatment. For the second approach, 10 μg each of the *Ac*PDO samples was incubated for 1 min in enzyme reaction buffer with (1) 1 mM GSH, (2) 2% sulfur flower, (3) both, and (4) 1 mM GSSH prepared by the first method described above (GSSH synthesis and quantification). The reaction was stopped by addition of 15 mM *N*-ethylmaleimide (NEM; Serva, Heidelberg, Germany; 5 mM for assay mixtures without S^0^). The samples were separated by SDS-PAGE using the sample buffer without reductant. The gels were stained with colloidal Coomassie Blue, the *Ac*PDO bands excised and sent to the MS facility for tryptic digestion and measurement. The mass spectra were evaluated using SCAFFOLD 4 (Proteome Software, Portland, OR, United States).

### Inhibition Studies

Inhibition studies were performed with IAA and NEM. Different approaches were followed for testing inhibitory effects. First, IAA and NEM were directly added to the enzyme reaction mixture at the final concentration given in the Section “Results” in order to verify the results obtained by Wang et al. (2014), since it remained unresolved whether the modifying reagents had bound to the cysteine residues of GSH, or of the protein, or both. In a different approach, the PDO (≈5 mg/ml) was incubated for 20 min with 3 mM NEM or 5 mM IAA followed by dialysis against 50 mM Tris/HCl buffer at pH 7.5 for 24 h (dialysis volumes: 2 × 300 ml; 1 × 400 ml) and subsequent measurement of the enzyme activity.

The reduction of putative disulfide bonds was performed using Tris(2-carboxyethyl)phosphine (TCEP; Sigma-Aldrich, Munich, Germany) in an anaerobic glove box (Coy; <0.5 ppm O_2_) to prevent instantaneous re-oxidation. Purified enzyme (5 mg) was dialyzed to remove desthiobiotin from the elution buffer as outlined above and applied to a 1 ml-Strep-Tactin Superflow column matrix. 3 ml of 3 mM TCEP followed by 3 ml of 3 mM IAA or 2 mM NEM were applied to the column-bound protein and incubated for 10 min. Afterwards, the modified enzyme was washed and eluted using the standard elution buffer (IBA). The elution fractions were removed from the glove box and activity assays were immediately performed under air.

### Protein Denaturation Experiments

Stock solutions of guanidinium chloride and urea were prepared in 150 mM Tris/HCl buffer, pH 8, and added to 1 mg/ml of purified enzyme to the final molarities given in the Section “Results.” After incubation at 25°C for 1 h, 200 μl of the mixtures were applied to a Superose 6 HR 10/30 gel permeation column equilibrated with the denaturant/buffer mixture used for enzyme denaturation. The column was developed with the same mixture at a flow rate of 0.5 ml/min.

## Results

### Enzyme Properties of the *Ac*PDO

The *pdo* gene was amplified from *Acidithiobacillus caldus* C-SH12 DNA and ligated with the expression vector pASK75 (Skerra, 1994) so that a C-terminal Strep-Tag was attached for purification to the resulting protein (*Ac*PDO). When the gene was expressed in *E. coli*, the average yield was 26.3 ± 20.6 mg of purified protein/l of LB medium (*n* = 18 preparations). The average iron content was 0.77 ± 0.1 nmol Fe/nmol of protein (*n* = 6 preparations each measured 3 times). SDS-PAGE showed a major protein band with an apparent molecular mass of 27 kDa, representing the *Ac*PDO monomer (Supplementary Figure S1A). Western hybridization with a horse radish peroxidase-coupled anti-strep-tag antibody and chromogenic signal detection resulted in a strong signal from the 27 kDa band and a weak signal from the minor band of ≈57 kDa (derived from *R*_f_ value) suggesting that the latter represents the dimeric state of the protein (Supplementary Figures S1A,B, S2A).

**FIGURE 2:**
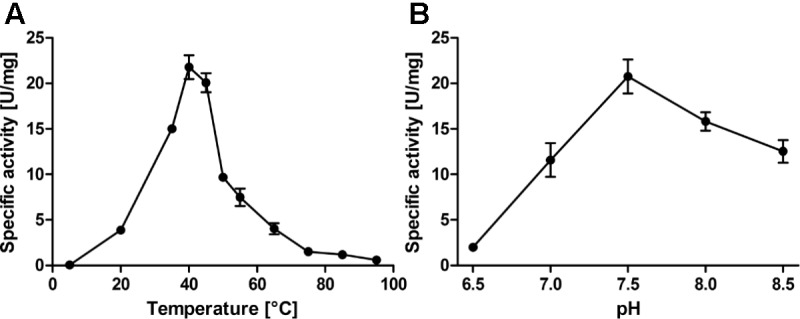
Temperature **(A)** and pH profiles **(B)** of the *Ac*PDO with 0.2 mM GSH and 2% sulfur and various amounts of wild type *Ac*PDO as appropriate (1–100 μg/ml). Error bars represent the standard deviation from triplicate measurements.

**FIGURE 3:**
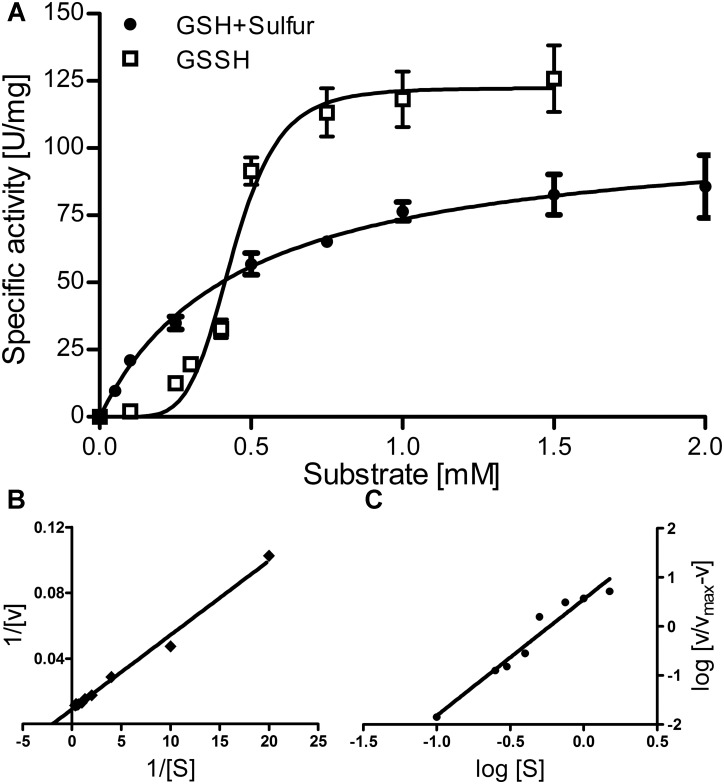
Enzyme kinetics of the *Ac*PDO. **(A)** activity vs. substrate concentration plot for GSH plus sulfur and GSSH; Error bars represent the standard deviations from triplicate measurements. **(B)** Lineweaver–Burk plot for GSH plus sulfur; **(C)** Hill plot for GSSH.

The assay for PDO enzyme activity was derived from a similar one used to measure sulfur oxygenase reductase activity (Rühl and Kletzin, 2017): The Tris-based assay buffer containing elemental sulfur, detergent and GSH was pre-warmed in a thermomixer. After addition of the enzyme and mixing, aliquots were taken every 10 s (50 s total assay time) and the reaction was stopped by mixing the aliquot with acid fuchsine solution prior to color development by addition of formaldehyde. Using this assay, average specific activities of 61.6 ± 3.5 U/mg protein were obtained at 40°C and pH 7.5 in the presence of 1 mM GSH (**Table 1**, **Figure 2** and Supplementary Figure S3; *n* = 18 independent protein preparations, with 39 measurements in total). The specific activity even increased to ≈85 U/mg in the presence of 2 mM GSH (**Figure 3**), however, either 1 mM or 0.2 mM GSH was used in routine experiments. Sulfite was the only reaction product up to 45°C. At 50°C and above, thiosulfate was also detected, which is the product of a non-enzymatic reaction of sulfite and excess sulfur at elevated temperature (Supplementary Figure S4; Roy and Trudinger, 1970; Kletzin, 1989; Rühl et al., 2017). The temperature span of *Ac*PDO activity was 90 K (**Figure 2A**; note that the activities in the temperature and pH curves were measured at the sub-optimal GSH concentration of 0.2 mM because this had been done before the final optimization of the enzyme assay was accomplished: in this case the total incubation time of the enzyme assay was 10 min with intervals of 2 min). The use of chemically synthesized GSSH (Supplementary Figure S5) resulted in specific activities of 118 ± 10 U/mg protein at 1 mM substrate (**Figure 3** and Supplementary Figure S2). When GSSH was synthesized by heating of GSH with sulfur, the resulting supernatant contained a mixture of non-reacted GSH and GSSH (not shown).

**Table 1:** Comparison of the activity measurements and kinetic constants obtained of PDOs from *Ac. caldus* C-SH12 and MTH-04.

*Ac. caldus* strain				C-SH12	MTH-04	MTH-04
Accession/locus tag				PRJEB24175	A5904_0790	A5904_0421
	Substrate	Co-substrate^a^	Unit			
Specific activity	1 mM GSH	2% S^0^	U/mg	61.6 ± 3.5	2.34^b^	n.r.^c^
	1 mM GSSH	–	U/mg	118 ± 10	n.r.	0.066
*V*_max_	1 mM GSH	2% S^0^	U/mg	111.5	n.r.	n.r.
	1 mM GSSH	–	U/mg	122.3	n.r.	n.r.
*K*_M_	GSH	2% S^0^	μM	≈500	n.r.	n.r.
	GSSH	–	μM	Not applicable^d^	298 ± 13	267 ± 31
*K*_cat_	GSH	2% S^0^	s^−1^	181	n.r.	n.r.
	GSSH	–	s^−1^	198	48.1	5.4
*K*_cat_/*K*_M_	GSH	2% S^0^	mM^−1^ s^−1^	361	n.r.	n.r.
	GSSH	–	–	Not applicable^d^	161.4	20.2
Hill coefficient	GSSH	–	–	2.3 ± 0.22	n.r.	n.r.
Reference				This work	Wang et al., 2014;	Wu et al., 2017
					Wu et al., 2017	

*^a^Both added in the enzyme assay mixture. ^b^0.8 mM GSH; 1 U of activity had been defined as 1 nmol sulfite formed per min, not 1 μmol (Wang et al., 2014). ^c^n.r., not reported. ^d^K_M_ was not calculated due to the sigmoidal curve (**Figure 3**).*

The Michaelis–Menten plot of GSH in the presence of excess sulfur followed a saturation curve up to the highest measured concentration (7.5 mM; **Figure 3A**, shown up to 2 mM). This and the corresponding Lineweaver–Burk plot resulted in a *K*_M_ value of ≈0.5 mM, a *V*_max_ of 111.5 U/mg protein, a *K*_cat_ of 181 s^−1^ and a specificity constant *K*_cat_/*K*_M_ of 361 s^−1^ mM^−1^ per subunit (**Figures 3A,B**). In contrast, GSSH gave a sigmoidal curve with a *V*_max_ of 122.3 U/mg protein at 1–1.5 mM GSSH suggesting positive cooperativity and a *K*_cat_ of 198 s^−1^. The Hill plot resulted in a straight line and the resulting Hill coefficient n_H_ was 2.3 ± 0.22 (**Figure 3C**), suggesting the presence of more than two subunits in the active holoenzyme.

**FIGURE 4:**
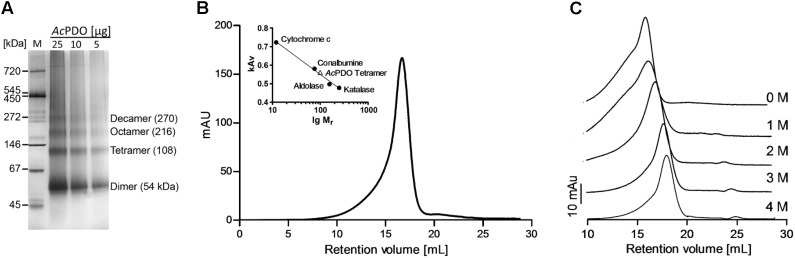
Native *Ac*PDO and effects of denaturants. **(A)** Non-denaturing 4–16% polyacrylamide gel with different amounts of *Ac*PDO; M, Native marker liquid mix for BN/CN (Serva, Heidelberg, Germany); **(B)** Gel permeation chromatography of freshly prepared *Ac*PDO (0.5 mg protein) with marker proteins; **(C)** Gel permeation chromatography of previously frozen *Ac*PDO (0.1 mg protein) in the presence of different concentrations of guanidinium hydrochloride.

The major band in non-denaturing polyacrylamide gels of the *Ac*PDO had an apparent molecular mass of 54 kDa presumably corresponding to the dimer. Higher oligomeric states were also visible presumably corresponding to the tetramer, octamer and decamer (apparent molecular masses: 111, 203, and 285 kDa; **Figure 4A** and Supplementary Figure S2B). Gel permeation chromatography (GPC) of freshly prepared *Ac*PDO showed a major elution peak corresponding to an apparent mol. mass of 99.4 ± 1.5 kDa (*n* = 3; **Figure 4B**), equivalent to 3.7 subunits suggesting a tetrameric state in solution. A long slope toward lower retention volumes was visible suggesting the presence of higher oligomers as seen in the non-denaturing gels. This effect was enhanced when previously frozen protein preparations were used, which resulted in an additional shoulder in the chromatogram with an apparent mol. mass of ≈380 kDa (**Figure 4C**). GPC of guanidinium hydrochloride-treated *Ac*PDO resulted in a step-wise increase in retention volumes with the concentration, which points to a disintegration into dimers at 2 M and into monomers at 3 M guanidinium (**Figure 4C**). In contrast, urea treatment of the *Ac*PDO did not shift the dominant GPC peak toward higher retention volumes even in the presence of 8 M urea (Supplementary Figure S6A), however, the 380 kDa shoulder vanished from before-frozen PDO preparations. The 54-kDa dimer band was resistant against disintegration by urea. The monomer band started to appear only at 4 M urea and above, however, the dimer band remained the dominant species even at a urea concentration of 8 M (Supplementary Figure S6B). A peak with a retention volume of about 22.73 ml not showing bands in an SDS gel (not shown) appeared corresponding to a molecular mass ≈0.9 kDa suggesting low-molecular weight compounds were responsible. Taken together, the *Ac*PDO seems to assume a tetrameric state in solution, which is resistant against denaturation with urea but not with guanidinium hydrochloride and SDS.

MALDI-TOF mass spectrometries of the as-isolated *Ac*PDO holoenzyme gave peaks centering at 27,067 mass units (MALDI-TOF; monomer; Supplementary Figure S7) and 54,148 mass units (dimer), whereas electrospray MS consistently gave 27,074 mass units, which almost fit the simulated spectrum (the calculated molecular weight is 27,077.39, average isotopic composition including Strep-tag). Minor peaks centering at 27,090 and 27,106 (Supplementary Figure S7) each differ by the value of one oxygen molecule suggesting different oxidation states. Similarly, the dimer had minor peaks ± 32–33 mass units (not shown).

### Fe-Binding Residues

**FIGURE 5:**
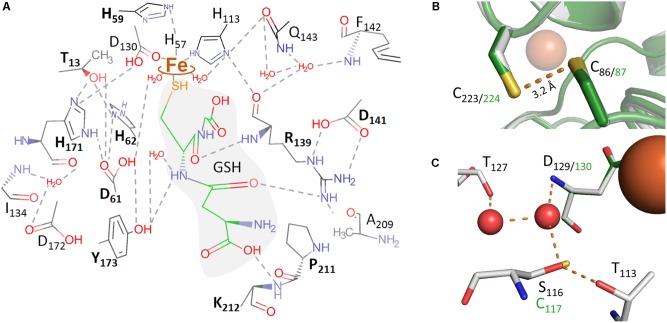
Molecular representations of the *Ac*PDO 3D model with glutathione from the *Pp*PDO structure (5VE5). **(A)** Theoretical model of GSH in the *Ac*PDO active site structure and predicted hydrogen bonds; boldface amino acid residues were mutagenized in this study. **(B)** C_86_ and C_223_ in the *Mx*PDO 3D structure (gray) and in the *Ac*PDO model (green). **(C)** Comparison of the secondary coordination sphere around D_129/130_ between the *Mx*PDO (gray) and the *Ac*PDO (green) originating from the S_116_ residue in the *Mx*PDO.

**FIGURE 6:**
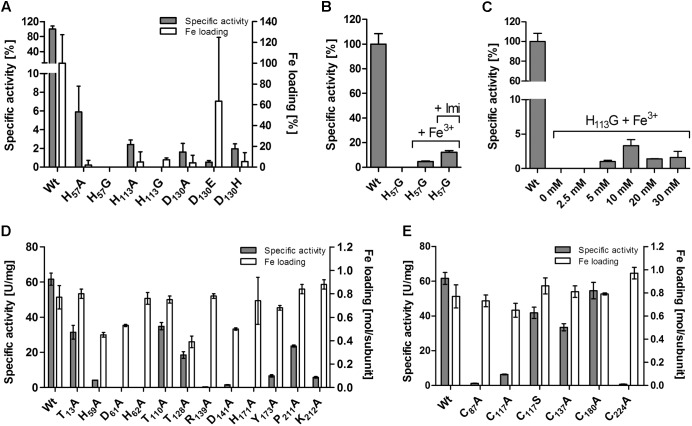
Effects of site-directed mutagenesis in the *Ac*PDO gene on the enzyme activity and iron content of the corresponding protein variants. **(A)** Variants of the iron ligands. **(B)** Reconstitution of the H_57_G variant with Fe and imidazole in the enzyme assay buffer. **(C)** Reconstitution of the H_113_G variant with Fe and increasing concentrations of imidazole. **(D)** Mutagenesis of substrate-binding site and hydrogen bond network. **(E)** Cysteine variants. Error bars represent the standard deviation from triplicate measurements.

Mutagenesis of the codons for putative iron ligands H_57_, H_113_, and D_130_ (**Figure 5A**) to alanine resulted in inactive enzyme except for H_57_A, which retained 6% residual activity and ≈0.1 Fe/subunit (**Figure 6A** and Supplementary Table S2). H_57_G and H_113_G variants were completely inactive and did not have any iron bound. The activity could be partially restored by the addition of imidazole and iron to the activity assays of the H_57_A/G variants but not of the H_113_A/G variants (**Figures 6B,C**). However, the imidazole/Fe binding was not strong enough to withstand dialysis for 24 h against buffer W since the dialyzed protein preparations lost their activity (not shown). The replacement of the D_130_ ligand with Ala, Glu or His resulted in low but measurable residual activities of 0.5–2%. D_130_A and D_130_H had low iron content, whereas the Fe loading of the D_130_E variant fluctuated considerably in different preparations (**Figure 6A**).

### Putative GSH-Binding Residues Around the Iron Site

A multiple sequence alignment of all PDO-like proteins with experimentally determined mononuclear iron in their active sites showed that the secondary structure elements could be overlaid reproducibly as observed earlier in a comparison of PDOs and glyoxalase II (Sattler et al., 2015; **Figure 1**; metallo-beta-lactamase type proteins with Zn^2+^ or dinuclear metal sites were excluded from the comparison). The most similar PDOs with an available 3D structure were the enzymes from *Myxococcus xanthus* (*Mx*PDO; 59% identity; PDB accession 4YSB; Sattler et al., 2015) and *Paraburkholderia phytofirmans* (*Pp*PDO; 49%; 5VE5; Motl et al., 2017; note that the organism was recently renamed; Sawana et al., 2014). The latter is fused C-terminally with a rhodanese domain. Structure prediction of the *Ac*PDO with the *Mx*PDO 3D structure as the template resulted in a tightly fitting molecular model with a root mean square deviation (r.m.s.d.) of 0.125 Å, whereas the r.m.s.d. with the PDO domain of the *Pp*PDO was 0.603 Å (Supplementary Figures S8A,B) in accordance with the pairwise sequence identities (PDB file is included in Supplementary Sequence File). The *Mx*PDO and the PDO domain of the *Pp*PDO could be superimposed to a, similarly, degree (r.m.s.d. = 0.586 Å; 51% identity). Therefore, the GSH coordinates of the *Pp*PDO were used for prediction of the GSH-binding residues in the *Ac*PDO, the hydrogen bond network and the mononuclear iron site.

Sattler et al. (2015) had also presented the 3D structure of the Type II PDO from *Pseudomonas putida*, again with bound GSH (4YSL). The comparison showed that two of the GSH-binding residues of the *Paraburkholderia* PDO were not conserved in the *Ps. putida* enzyme (R_190_ and K_212_ in *Ac*PDO numbering; **Figure 1**), while two others were conserved (R_139_ and Y_173_). R_139_ is located in the center of the active site: the backbone oxygen is in H-bonding distance to the Nδ-atom of the iron ligand H_113_ (Supplementary Figures S9A,B). The ε- and the η1-nitrogen atoms are in H-bonding distances to the side-chain carboxyl oxygens of D_141_ (ionic pair) and the η2-nitrogen seems to form H-bonds to the backbone oxygen of P_211_ (**Figure 5A**). Expectably, R_139_A and D_141_A variants had residual activities of only 0.4 and 1.5%, respectively (**Figure 6D** and Supplementary Table S2), consistent with similar observations of mutations of the homologous R_163_ and D_165_ sites of the hETHE1 (Tiranti et al., 2006; Henriques et al., 2014). In addition, the homologous residue R_142_ of the *Pp*PDO forms an H-bond to the εO_1_ atom of the glutamyl side chain of the GSH (Motl et al., 2017).

Among the other predicted GSH-binding residues, Y_173_A and K_212_A variants each retained ≈10% residual activity whereas the neighboring P_211_A variant retained 39%, suggesting that K_212_ rather than P_211_ is the interaction partner with GSH (**Figures 5A**, **6D** and Supplementary Table S2). The experimentally seen interaction partner is the homologous residue K_216_ of the *Pp*PDO (Motl et al., 2017).

### Hydrogen Bonding Network

Secondary coordination sphere residues include the conserved T_56_ (**Figure 1**) predicted to form a hydrogen bond to Nδ of the iron ligand H_57_, and H_171_ predicted to form H-bonds to the Oδ_2_ atom of the iron ligand D_130_ and to T_13_ (Supplementary Figures S9C,D). The Nε atom of H_171_ is also hydrogen-bonded to the side chain of D_61_, which in turn forms an H-bond to H_62_ (**Figures 5A,C**). H_59_ – homologous to the hETHE1-H_81_ residue (Lin et al., 2016)– bridges H_57_ and H_62_ and makes an H-bond to D_30_. The H_59_A mutant had 6.6% residual activity, whereas the T_56_A variant could not be purified at all and no protein was visible in SDS gels of the expression strain, even by Western hybridization. H_171_A, D_61_A, and H_62_A were produced in *E. coli* but did not have activity above the detection level (≤0.05 U/mg with 100 μg/ml of enzyme in the assay; **Figure 6D** and Supplementary Table S2), consistent with their central positions in the active site H-bond networks. The T_128_A and T_110_A variants retained 39 and 57% activity, respectively (**Figure 6D** and Supplementary Table S2). The T_128_ side chain oxygen is in H-bonding distance to the backbone nitrogens of D_130_ and A_131_ so that T_128_ seems to stabilize the loop around the iron ligand (**Figures 5A,C**).

### Cysteine Residues and Inhibition of the *Ac*PDO

The multiple alignment (**Figure 1**) also showed that cysteine residues vary in numbers in the different PDOs and that they are not generally conserved, not even the essential C_247_/C_235_ of the human and *Arabidopsis* PDOs, respectively, which are present as cysteine sulfinic acid residues in the 3D structures (**Figure 1**; McCoy et al., 2006b; Pettinati et al., 2015; Jung et al., 2016). The *Ac*PDO amino acid sequence comprises 5 cysteine residues, two of which (C_87_ and C_224_) are predicted to be in close proximity at the surface of the protein (**Figure 5B** and Supplementary Figure S10). In the *Mx*PDO, the corresponding cysteines C_86_ and C_223_ show an S–S distance of 3.5 Å (Sattler et al., 2015), too far for a disulfide bridge (2–2.1 Å) although slight structural rearrangements would be sufficient to change that (**Figure 5B**). Mutagenesis of either the two *Ac*PDO C_87_ and C_224_ residues to alanine resulted in a drop of enzyme activity to low levels (2.2 and 1.3%, respectively; **Figure 6E**).

**FIGURE 7:**
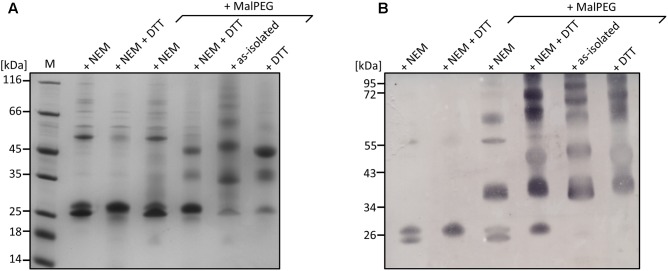
Analysis of *Ac*PDO using a MalPEG gel shift assay. **(A)** Coomassie-stained 10% Tris-tricine gel of the *Ac*PDO wild type (10 μg/lane). **(B)** Western analysis using StrepMAP-Classic HRP-conjugated antibody. M, Marker in kiloDalton, NEM, sample derivatized with *N*-ethylmaleimide, DTT, sample reduced with dithiothreitol.

Gel shift assays with the as-isolated and NEM-treated wild-type enzyme showed two monomer bands with apparent masses of 26–28 kDa in Coomassie-stained gels and after Western blotting with an α-Strep-tag antibody (**Figure 7**) beside the dimer band of ≈54 kDa. The smaller of the two monomer bands disappeared upon reduction with DTT and was also not visible with the C_87_A and C_224_A variants (**Figure 7** and Supplementary Figures S1C–F), similar to SDS-gels with reducing sample buffer (Supplementary Figures S1A,B) suggesting that the non-reduced protein adopts two confirmations running differently in SDS gels. The smaller band was, however, visible in the non-reduced wild type enzyme after incubation with methoxypolyethylene glycol maleimide-5000 (MalPEG) but not in the variants indicating protection against PEGylation. Up to five bands with higher masses appeared differing by ≈10 kDa each and indicating that all 5 cysteines are at least partially accessible to PEGylation (**Figure 7**; the shift by 10 kDa is typical for MalPEG-5000-treated proteins in Tris-tricine gels; Venceslau et al., 2013). There were also small but consistent differences in the apparent masses of the 35 and 45 kDa bands of the DTT-reduced versus the as-isolated proteins similar to the unmodified monomer band (single and double-PEGylated *Ac*PDO, respectively). We interpret the combined results as an indication for the presence of a disulfide bond between C_87_ and C_224_ in a fraction but not in all of the molecules of the *Ac*PDO protein preparation.

Among the other cysteines, the C_117_A variant had 12% residual activity, whereas the activities of the C_137_A and C_180_A variants were less affected (54 and 89%, respectively). C_117_ is replaced by a serine in the *Mx*PDO, which is at the center of a network of H-bonds and water molecules around the histidine ligands presumably stabilizing the spatial arrangement of the iron site but not being actively involved in catalysis (**Figure 5C**). A C_117_S variant had 68% of wild type activity supporting this hypothesis (**Figure 6E** and Supplementary Table S2).

MALDI-TOF mass fingerprinting was performed of as-isolated *Ac*PDO, sulfur- and GSSH-incubated and of the dithiothreitol-reduced protein with or without alkylation with iodoacetamide (IAA) or *N*-ethylmaleimide (NEM). Most cysteine-containing mass fragments were found as thiols, regardless of the oxidation state of the enzyme. Minor counts of cysteine dioxides and trioxides were identified and no direct evidence was found of cysteine persulfides (data not shown). In contrast, cysteine-GSH adducts were identified after the enzyme was incubated with GSSH. C_87_ contained high proportions of these adducts compared to NEM derivatization (>50%; Supplementary Figure S11). Lower counts were present at C_224_ and C_137_, however, C_224_ was represented in the mass spectra only by an incompletely digested octadecapeptide, whereas the fully digested tripeptide was below the detection range of the instrument. The C_87_A and C_224_A variants had the expected mass differences of the respective fragments (-32 mass units) whereas the remaining cysteine retained partial glutathionylation (Supplementary Figure S11). C_117_ and C_180_ showed no more than two glutathionylation signals in total (not shown) and were therefore considered inactive in this respect whereas intermediate counts and percentages (<40%) were recorded for C_137_, which also lies closed to the surface according to the structural model.

**FIGURE 8:**
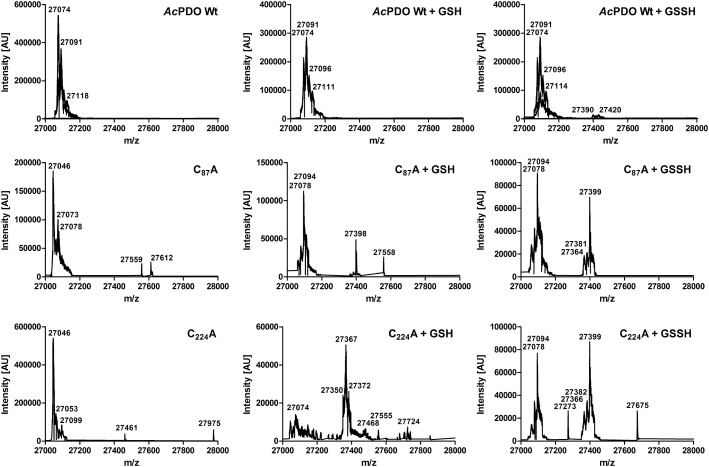
ESI mass spectra of the *Ac*PDO wild type, the C_87_A and the C_224_A variants of the as-isolated proteins and after incubation with GSH and GSSH, respectively.

ESI MS of the wild type *Ac*PDO holoenzyme resulted in major peaks of 27,074 and 27,091 mass units close to the expected sizes of the unmodified enzymes and to a single oxidation event (+16; **Figure 8** and Supplementary Figure S7). A broad range of minor peaks is present at 27,390–27,420 mass units in the wild type. The difference is slightly higher than the mass of GSH (305 mass units) suggesting partial oxidation and/or the presence of attached sodium ions. A different pattern was observed with the C_87_A and C_224_A variants. The major peak of 27,046 mass units for the unmodified enzyme was shifted to 27,078/27,094 mass units in either case after incubation with GSH or GSSH suggesting either persulfuration or double/triple oxidation. In addition, the 27,398-9 peaks suggest glutathionylation due to the 305-mass-unit difference to the 27,094 peak. Only C_224_A incubated with GSH gave a more complex pattern with the major peak corresponding to a single-oxidized GSH adduct. Control measurements with TCEP-treated wild type enzyme could not be interpreted as they resulted in undefined fragmentation patterns of the whole protein (not shown). In conclusion, both the MALDI fingerprint and the whole-enzyme MS analysis and its cysteine variants gave evidence for covalent glutathionylation.

**FIGURE 9:**
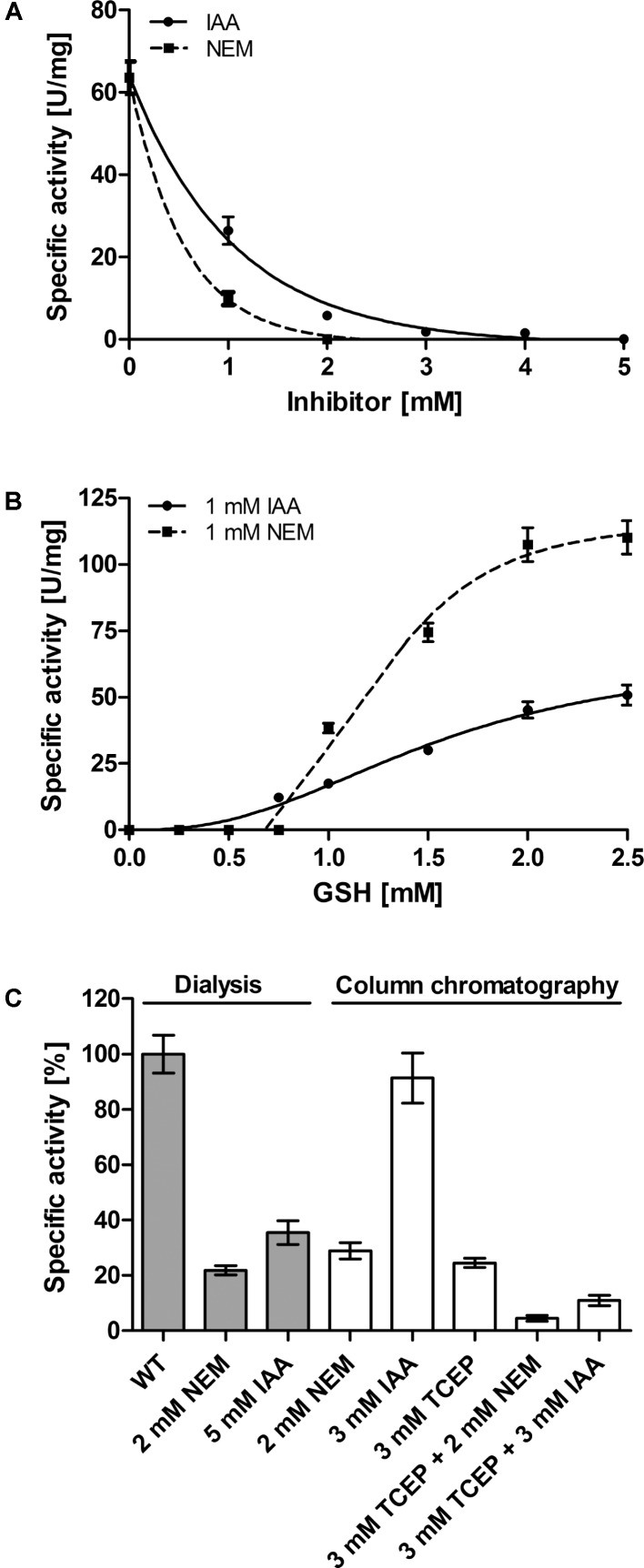
Effect of Inhibitors on the *Ac*PDO. **(A)** Residual PDO activity with 1 mM GSH, sulfur and *N*-Ethylmaleimide (NEM) or iodoacetamide (IAA) after adding the substance directly to the enzyme assay mixture. **(B)** Same as in **A** only that the GSH concentration was varied. **(C)** Residual PDO activity after pre-incubation of the *Ac*PDO with NEM, IAA, or Tris(2-carboxyethyl)phosphine (TCEP). Error bars represent the standard deviation from triplicate measurements.

Wang et al. (2014) had reported that NEM inhibits the *Ac*PDO almost completely at a concentration of 1 mM but they had not addressed the question whether the substance inactivated the thiol-containing substrate GSH or the enzyme or both. When we used a similar approach with 1 mM GSH and excess sulfur as substrates, we found 16% residual PDO activity in the presence of 1 mM NEM and no activity at 2 mM, the corresponding residual activities were 42 and 9% for IAA, respectively, while total inhibition required 5 mM IAA (**Figure 9A**). When the inhibitor concentration was kept constant at 1 mM and the co-substrate GSH was titrated, the PDO activity was absent at GSH concentration below 0.75–1 mM. Increasing GSH concentrations restored wild type levels, showing that the inhibitors do not specifically bind to the enzyme but to all available thiols (**Figure 9B**). When we pre-incubated the *Ac*PDO with NEM and removed excess inhibitor by dialysis or Strep-Tag affinity chromatography, the residual activities were 22–29% compared to the inhibitor-free but likewise treated control (**Figure 9C**). The enzyme treated with 5 mM IAA showed 36% activity (removal of the inhibitor by dialysis) or 91% with 3 mM IAA (column purification; **Figure 9C**). The results show that inhibition by NEM and IAA is at least partially due to GSH modification and that the inhibition is not permanent.

In order to determine whether the redox state of the *Ac*PDO is important for catalysis, the protein was reduced in an anaerobic glove box with Tris(2-carboxyethyl)phosphine (TCEP) and the reductant was removed by Strep-Tag affinity chromatography under anaerobic conditions. The residual activity was ≈25% compared to non-reduced protein when measured under standard aerobic conditions immediately after chromatography. When incubating the TCEP-reduced enzyme additionally with 2 mM NEM or 3 mM IAA, the residual activity was between 5 and 10% compared to the untreated and unreduced protein (**Figure 9C**).

### Melting Points of *Ac*PDO and Its Variants

**FIGURE 10:**
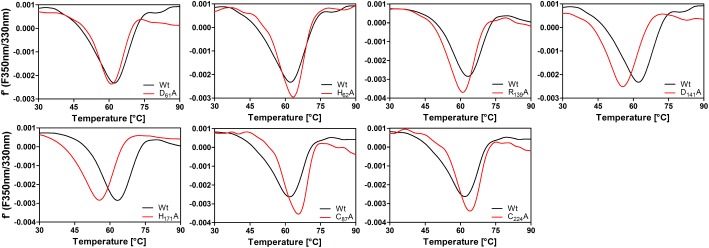
First derivative plots of differential scanning fluorimetry (tryptophane/tyrosine fluorescence) of *Ac*PDO variants (1 mg/ml) measured in 100 mM Tris buffer, pH 8.0, at a heating rate of 1 K/min and comparison with wild type.

Differential scanning fluorimetry (DSF) of the intrinsic tryptophane fluorescence showed a mean denaturation temperature of 63 ± 1.3°C at a heating rate of 1°C/min (*n* = 5 preps, each measured 3 times; **Figure 10** and Supplementary Figure S12). The results of the temperature curve combined with the melting point suggest that the activity at temperatures higher than 65–70°C was detectable because of the short overall reaction time during the activity assay, so that the enzyme might be slow in its denaturation kinetics. The melting points of the enzymatically inactive D_61_A and H_62_A variants were similar to the wild type (**Figure 10**). In contrast, the low-activity variants R_139_A, D_141_A, and H_171_A had markedly reduced melting points of 60.7 ± 0.8°C, 55.7 ± 0.2°C, and 54.9 + 0.7°C, respectively, showing that the predicted salt bridge between R_139_ and D_141_ is important for stability as are the hydrogen bonds of H_171_. DSF of the two *Ac*PDO C_87_A and C_224_A variants gave melting points of 63.3 ± 1.4°C and 63.3 ± 0.05°C, respectively (**Figure 10**), showing that the overall stability of the protein is not affected by these mutations.

## Discussion

We report here an analysis of the biochemical properties of the ETHE1-like persulfide dioxygenase from the acidithermophilic bacterium *Acidithiobacillus caldus* C-SH12 (T_opt_ = 45°C, pH_opt_ = 2–2.5; Hallberg and Lindström, 1994). The closely related *Ac. caldus* strain MTH-04 contains three *pdo*-like genes, whereas only two of the heterologously produced proteins showed PDO activity (Wu et al., 2017). They are encoded by the ORFs A5904_0421 (termed SDO1 by the authors) and A5904_0790 (SDO2, mutual sequence identity 33%). The SDO2 protein is 99% identical to the PDO described here. The deletion or overexpression of both *sdo* genes did not affect the growth properties of MTH-04 on elemental sulfur significantly (Wu et al., 2017) suggesting that other sulfur-oxidizing enzymes like the sulfur oxygenase reductase (Janosch et al., 2009, 2015) might be more important. A deletion of the *sdo1* gene, however, resulted in a complete inability of MTH-04 to grow on tetrathionate (Wu et al., 2017). Similarly, Wang et al. (2014) had deleted the SDO2-homologous *pdo-*gene from the genome of the related bioleaching bacterium *Ac. ferrooxidans*, again showing that the PDO was not the only enzyme responsible for chemolithoautotrophic growth on sulfur in both bacteria.

Biochemical characterization of the two MTH-04 PDOs showed that both use GSSH as substrate with a comparable *K*_M_ but a *K*_cat_ differing ca. ninefold (**Table 1**; Wu et al., 2017). The maximal specific activity of SDO2 was reported to be 2,336 mU/mg protein with sulfur and GSH at pH 8.0 (Wang et al., 2014), whereas SDO1 had 66 mU with GSSH (Wu et al., 2017). We obtained a ≈25-fold higher value with GSH + S^0^ as substrates compared to the homologous SDO2. Even higher values were obtained with GSSH and an optimized protocol at 40–45°C and pH 7.5 (**Table 1**). The difference was that we used a pre-warmed reaction buffer and shorter incubation times (50 s in 10 s intervals) and that we started the reaction by addition of the enzyme.

The short reaction times of the enzyme assay might also explain the maximal temperature of activity being considerably higher than the melting point of the protein. We had observed a similar effect in a sulfur oxygenase reductase from the mesophilic bacterium *Thioalkalivibrio paradoxus* (*Tp*SOR), with an optimal activity and a melting point each of 80°C and a temperature maximum of about 98°C (Rühl et al., 2017). The multimeric SORs (24 identical subunits) are unrelated to PDOs but they also contain a mononuclear iron site with a 2-His-1-carboxylate facial triad in the active site (Urich et al., 2006).

### The *Ac*PDO Is a Homotetramer

The *Ac*PDO seems to be homotetrameric in solution as evidenced both by non-denaturing gels and gel permeation chromatography (**Figure 4** and Supplementary Figure S6) and resistant against denaturation with urea but not against guanidinium hydrochloride. The quaternary structure is consistent with the sigmoidal curve for GSSH in the Michaelis–Menten plot (**Figure 3**) and the Hill coefficient n_H_ of 2.3 suggesting positive cooperativity. Since we found that GS(S)H binds to cysteine residues as well as to the iron site, the sigmoidal curve might also be a result of the substrate binding at two different places of the *Ac*PDO (see below, C_87_ and C_224_ are essential residues). A similar sigmoidal dependency of activity on the GSSH concentration was observed measuring the O_2_-consumption rate in the PDO from the echiuran worm *Urechis unicinctus*, however, the enzyme was not analyzed with respect to cooperativity (see figure 9 in Zhang et al., 2013). The difference to GSH plus sulfur as substrates might also be explained by the unknown kinetics of the non-enzymatic addition of S^0^ to the reactive GSH thiol in the assay mixture, a reaction, which had led to the original name of “sulfur dioxygenase” for this type of enzymes (Rohwerder and Sand, 2003; for a review of the general principle, see Toohey, 2011).

### Homology Model

The *Ac*PDO had the highest similarity with the *Mx*PDO (PDB accession 4YSB) so it was used to generate a homology model. Both proteins could be superimposed with little deviation (Supplementary Figure S8) and with the PDO domain of the *Pp*PDO (5VE5). We transferred the coordinates of the GSH moiety of the *Pp*PDO to the *Ac*PDO model in order to analyze putative contact sites. The rhodanese domain of the *Pp*PDO did not interfere with the access to the active site cleft of the PDO domain (Motl et al., 2017) and also not with access to the conserved cysteines C_224/228_.

The problem, which of the residues of the *Ac*PDO and other homologs are important for catalysis, breaks down to three partially interconnected issues: (i) the iron ligands, (ii) the secondary coordination sphere and the hydrogen bonding network, including the GSH/GSSH-binding site, and (iii) the role and importance of the cysteines.

### Iron Ligands Are (Almost) Irreplaceable

It is obvious that the enzyme loses activity when the iron and its ligands are missing. The mononuclear iron-containing mono- or dioxygenases typically activate dioxygen by initial reduction to an iron-bound peroxide with varying oxidation states of the metal (Kal and Que, 2017). The three water ligands are displaced when the substrates are bound, whereas the amino acid ligands, two histidine and one aspartate, remain in place. It was surprising to see that mutagenesis of the iron ligands to alanine – in contrast to glycine – did not completely inactivate the enzyme activity and residual but low amounts of iron were present so that a weak but specific iron binding was possible with only two ligands (**Figure 6A**). We saw the same effect in the triple-His variant mimicking the iron site in the cysteine dioxygenases (McCoy et al., 2006a; Simmons et al., 2006). Fe loading seemed to be higher in the D_130_E variant, although the enzyme activity remained low. The H_57_A variant was least affected but the residual activity did not exceed 5–6% of the wild type. The activity of the glycine but not of the alanine variants of H_57_ and H_113_ could be partially restored by addition of imidazole and iron to the activity assay, however, both compounds did not bind stably to the enzyme (**Figures 6B,C**).

### Central Residues in the H-Bond Network Are Crucial for Enzyme Activity

The GSH/GSSH-binding residues had received some attention in the literature pertaining the human ETHE1 since R_163_ (hETHE1 numbering; **Figure 1**) is a hotspot for variations with the propensity to cause disease (Henriques et al., 2014). The backbone oxygen of R_163_ is also predicted to be part of the secondary coordination sphere (**Figure 5A**). Therefore, it is not surprising that the *Ac*PDO R_139_A-variant has low but not zero activity, similar to D_141_A, which forms an ionic pair with R_139_ (**Figure 5A**). Both alanine variants but especially D_141_A have reduced melting points (**Figure 10**). R_163_/R_139_ is one of only two GSH-binding residues conserved in all three types of PDO, the other being Y_173_ (Y_197_ in hETHE1, **Figure 1** and Supplementary Sequences). R_190_ and K_212_ (*Ac*PDO numbering) are replaced by the non-homologous R_250_ and R_253_ residues in the *Pseudomonas putida* PDO structure (**Figure 1**; Sattler et al., 2015) but they are conserved in Type I enzymes together with P_211_ (**Figure 1** and Supplementary Sequences). The residual activities of these variants were ≈10% for Y_173_A and K_212_A, while P_211_A retained about 38% showing that other residues can compensate the functional losses at least partially.

The region between T_56_ and H_62_ forms a short amino acid motif (consensus T-H-hydrophobic-H-A-D-H-hydrophobic-T/S; **Figure 1**), which is conserved in Type I and Type II enzymes, while Type III enzymes have the third His replaced by a phenylalanine or tyrosine (Supplementary Sequences). This motif is slightly longer than defined by Sattler et al. (2015).

T_56_ is a secondary coordination sphere residue, H_57_ an iron ligand, while D_61_ and H_62_ form an ionic pair that is connected to H_171_ (**Figure 5A**). H_59_ seems to stabilize the loop formed by these residues and was considered to be important for persulfide binding by Lin et al. (2016). All of mentioned residues are important for a functional enzyme since no activity was measured in the alanine variants with exception of H_59_A, which had 6.6% activity, while the T_56_A variant could not be produced at all. Moreover, H_171_A had a reduced melting point (**Figure 10**). The center of this H-bond network is formed by R_139_/D_141_ and H_171_, however, it extends far beyond the direct vicinity of the iron and GSH-binding sites.

### C_87_ and C_224_ Are Essential Residues

Cysteines are important for enzyme activity of the PDOs (Jung et al., 2016) in spite of little conservation in their respective positions. C_117_ and C_137_ are the only cysteines located near the iron site. Of these, only C_117_ is conserved in most bacterial PDOs albeit replaced by serine in the *Mx*PDO and some other Type I enzymes (**Figures 1**, **5C** and Supplementary Sequences; Sattler et al., 2015). The C_117_S replacement had activities close to wild type while the activity of the alanine variant dropped to about 10%. The Cys/Ser moiety seems to stabilize one of the loops around the iron site and is therefore considered an important part in the H-bond network. C_137_ and C_180_ did not seem to play an important role since the alanine variants were less affected in activity.

The *Mx*PDO comprises only two cysteine residues, both lying in close vicinity at the surface of each subunit but not in disulfide bond distance (**Figure 1**; Sattler et al., 2015). This suggests that the homologous *Ac*PDO residues C_87_ and C_224_ assume similar positions. C_224_ and its hETHE1 homolog C_247_ seem to be the only cysteines strictly conserved in the Type I enzymes and the only one found to be essential in hETHE1 (Jung et al., 2016). An alignment of the enzymes from the dataset used by Xia et al. (2017) confirmed this conclusion (Supplementary Sequences). In contrast, C_87_, like C_224_ found to be essential in this study, is not only missing from the PDOs of humans and *Arabidopsis* but also of several other bacteria. Non-reducing SDS-PAGE after NEM treatment showed a double band for the *Ac*PDO monomer, which was condensed to a single band after DTT treatment (**Figure 1**). The derivatization with MalPEG of the as-isolated protein showed five bands shifted each by ≈10 kDa, however, the smaller of the two monomer bands was still present (**Figure 1**). A similar shift was seen with the single and double-derivatized bands after MalPEG treatment with and without DTT, whereas NEM efficiently blocked PEGylation. These results suggest (1) that a C_87_ and C_224_ form a disulfide bond in parts of the as-isolated protein molecules but not in all of them and (2) that the oxidized protein is more compact and migrates faster in the SDS gel, regardless whether or not it was PEGylated. Strong and multiple PEGylation signals in the as-isolated protein without NEM pretreatment suggest a stepwise modification of all five cysteines (the Western hybridization signals were stronger than the Coomassie-stained bands but the signals were amplified by the peroxidase reaction and are not quantitative, **Figure 7** and Supplementary Figures S1D,F). The C_87_A and C_224_A variants shifted to high masses indicating modification saturation and suggesting a better accessibility of the otherwise partially protected cysteines in the interior of the protein (Supplementary Figures S1C–F). The reason might be same that makes the wild type enzyme move faster in the SDS gel: The α-helix at the C-terminus might be released to become more flexible around a rigid core of the protein. The latter conclusion might explain the melting points, which did not change in the two cysteine variants compared to the wild type.

Disulfides in proteins produced in the *E. coli* cytoplasm are not uncommon: Numerous proteins with intact disulfide can be found in the PDB or have been studied with other methods (e.g., thioredoxin, 3DIE, Garcia-Pino et al., 2009; artificial disulfide peptides, Pu et al., 2017; human Zn-Cu superoxide dismutase, Mercatelli et al., 2016). The fraction of oxidized versus reduced disulfides in the cytoplasm of *E. coli* and other cells depends on a combination of the actual reduction potential of the disulfide in question, the average reduction potential of the cytoplasm (*E. coli* BL21: −260 mV; range: −235–305 mV; Zhang et al., 2014), the ratio of redox mediators (mostly from the GSH/GSSG couple; E°′ ≈-250 ± 20 mV; see Gorin et al., 1975 and references therein) and the levels of formation of cysteine sulfenic acid in proteins by endogenous H_2_O_2_ and cytoplasmic thiol peroxidases (Flohe, 2013; Mercatelli et al., 2016). Disulfides will then form spontaneously from the reaction between thiol and sulfenic acid (Dalle-Donne et al., 2009; Winterbourn, 2015). Thioredoxin and glutaredoxin act as antagonists, they reduce cytoplasmic disulfides in a manner depending on the levels of NADPH and the respective reductases. From these considerations, the reduction potential of the *Ac*PDO disulfide should be in the same range, so that the protein is observed both in what we interpret as the oxidized and reduced forms (**Figure 7**).

C_87_, C_224_, and C_137_ were glutathionylated following the incubation of the enzyme with GSSH and, in the case of C_87_, also with GSH + S^0^ to a low degree (Supplementary Figure S11). ESI MS of the holoenzyme resulted in glutathione adducts predominantly of the C_87_A and C_224_A variants and to a lower degree in the wild type (**Figure 8**). Glutathionylation of either of the two disulfide-forming residues might be explained as the result of a thiol:disulfide exchange with GSSH. In contrast, glutathionylation of the free thiols in the C_87_A and C_224_A variants might be the result of other effects: (1) thiol:disulfide exchange with GSSH or (2) addition of GSH to a cysteine sulfenic acid residue (Dalle-Donne et al., 2009). Oxidation events are suggested by the shift of the 27,046 mass-unit peak of the C_87_A and C_224_A variants to 27,078 and 27,094 mass units even in the presence of GSH/GSSH, however, histidine, tryptophane, or methionine oxidation had been frequent in the MALDI MS fingerprints (not shown), therefore this might also be the effect of better accessibility of the residues in the absence of the disulfide. Glutathionylation often protects sensitive cysteine residues against the mostly irreversible oxidation to sulfinic and sulfonic acid species (the only exception being the peroxiredoxin – sulfiredoxin system; reviewed by Chae et al., 2012) and we expect this to be the case with the *Ac*PDO as well.

The low activity of the C_87_A and C_224_A variants points to an important role in catalysis and/or protein stability. Jung et al. (2016) had mutagenized the C_224_ homolog of the human enzyme to serine and had observed a similar decrease in activity. The authors had also shown that cysteines are modified by additional sulfane moieties, interpreted as protein-bound polysulfides. The method – chemical modification of the protein followed by gel shift assays – could, however, not distinguish between cysteine-bound sulfane sulfur, disulfide bridges or a putative cysteine S-glutathionylation. Additionally, the reduction with TCEP led to a decrease of activity of the *Ac*PDO to ≈25% and further derivatization of the reduced protein with NEM reduced activity to a level below each of the agents alone, suggesting that reduction of cysteines might render the whole protein more sensitive to the inhibitor and that breaking of the disulfide disfavors the enzyme reaction. TCEP has a reduction potential of about −290 mV and it is known as a non-sulfur-based disulfide-reducing agent (Pullela et al., 2006; Peng et al., 2013), however, it might also reduce the iron site. Taken together, the results suggest that the cysteines, disulfide formation and/or glutathionylation are essential for the *Ac*PDO although the results might have to be confirmed by cysteine-to-serine variants in order to exclude that neither hydrogen bonding effects nor the increased hydrophobicity of alanine are responsible.

## Conclusion

The results presented here point to a high importance of the hydrogen-bonding network around the iron site for substrate binding and catalysis in the *Ac*PDO. They also show, together with the results by Jung et al. (2016), that not only C_224_ (and its homologs in other PDOs) is important but also C_87_. C_224_ and C_87_ seem to provide a disulfide bond of yet unknown function, however, the decrease of enzyme activity upon reduction or mutagenesis point to a stabilization of the protein and of its substrate binding site by the disulfide. A direct participation in the reaction mechanism seems possible but unlikely since the cysteines are located in considerable distance to the iron site. Nothing is known about a putative interaction of these two sites and cannot be inferred from the subunit arrangements in the existing crystal structures (McCoy et al., 2006b; Pettinati et al., 2015; Sattler et al., 2015; Motl et al., 2017). The corresponding C_247_ residue was partially oxidized and present as sulfinic acid in the human and *Arabidopsis thaliana* PDOs (McCoy et al., 2006b; Pettinati et al., 2015) but it is unresolved whether this form represents the active enzyme or whether this is an oxidation product normally prevented by modification of the thiol (Jung et al., 2016).

S-glutathionylation of surface-exposed cysteines was seen here independently with two different mass spectrometric methods. Combined with the likelihood of the disulfide bond, the question remains whether these results represent an artifact, an integral part of the reaction mechanism of the PDOs, a protective mechanism against thiol oxidation or a structural feature. The unchanged melting points of the C_87_A and C_224_A variants speak against the latter hypothesis. Only the active site pocket around the iron was so far shown to bind GSH (Sattler et al., 2015; Jung et al., 2016; Motl et al., 2017). The hypothesis of low-molecular-weight thiols, glutathionylation and disulfide bond formation as protective mechanisms against uncontrolled thiol oxidation, however, is supported by literature data showing that this is a common mechanism in different proteins of numerous (micro-) organisms (e.g., Dalle-Donne et al., 2009; Mailloux and Willmore, 2014; Loi et al., 2015). It therefore seems probable that the cysteine S-glutathionylation, together with the putative disulfide bridge, serves as protection against (ir-) reversible oxidative damage, which is accompanied by a strong impairment of the enzyme activity, as shown by the mutation analyses of the cysteine residues in this study.

## Author Contributions

PR constructed most of the mutants and conducted most of the experiments, analyzed the results, prepared almost all the figures, and wrote parts of the introduction and the experimental procedures. PH, DS, and JB each constructed some of the mutants and performed some of the activity assays. AK wrote most of the main text and conceived the idea for the project.

## Conflict of Interest Statement

The authors declare that the research was conducted in the absence of any commercial or financial relationships that could be construed as a potential conflict of interest.

## Footnotes

1 https://www.dsmz.de/catalogues/details/culture/DSM-9466

2 https://www.chemie.tu-darmstadt.de/massenspektrometrie/masse/service_1/auftrag.de.jsp

3 http://ms-service.uni-hohenheim.de/
