# Supplementary material for: Persulfide Dioxygenase From Acidithiobacillus caldus: Variable Roles of Cysteine Residues and Hydrogen Bond Networks of the Active Site
Source: Front Microbiol. 2018 Jul 20;9:1610. doi: 10.3389/fmicb.2018.01610 (PMC6060420; doi:10.3389/fmicb.2018.01610)
Supplement: Supplementary file 1 [file Data_Sheet_1.pdf]

Persulfide dioxygenase from *Acidithiobacillus caldus*: Cysteine glutathionylation and identification of essential active site residues by mutation analysis

Patrick Rühl[1], Patrick Haas[1], Dominik Seipel[1], Jan Becker[1], and Arnulf Kletzin[1*]

[1]Department of Biology, Sulfur Biochemistry and Microbial Bioenergetics, Technische Universität Darmstadt, Schnittspahnstraße 10, 64287 Darmstadt, Germany

***Correspondence:**
Dr. Arnulf Kletzin, Fachbereich Biologie, Technische Universität Darmstadt, Schnittspahnstraße 10, 64287 Darmstadt, Germany, Telephone: (+49) 6151 16-23682, FAX: (+49) 6151 16-23672
Kletzin@bio.tu-darmstadt.de

**Contents**	**Page**
**Table S1:** Oligonucleotides used in this study	S-2
**Table S2:** Overview of specific activities of wild type *Acidithiobacillus caldus* PDO (*Ac*PDO) and its variants, iron content, numbers of preparations including total number of assays and T_m values	S-3
**Figure S1.** SDS Gel, MalPEG gel shift assays and Western analyses of the wild type and C_{87} and C_{224} variants of the *Acidithiobacillus caldus* PDO	S-4
**Figure S2.** Calibration plot of R_f values of denaturing and non-denaturing PAGE	S-5
**Figure S3.** Activity profile of the *Ac*PDO	S-5
**Figure S4.** Formation of sulfite and thiosulfate during the enzyme reaction of the *Ac*PDO.	S-6
**Figure S5.** Mass spectra of GSH and chemically synthesized GSSH	S-6
**Figure S6.** Urea denaturation experiments of wild type *Ac*PDO	S-7
**Figure S7.** Mass spectra of the *Ac*PDO holoenzyme	S-8
**Figure S8.** Comparison of the *Ac*PDO model with 3D structures of *Myxococcus xanthus* and *Paraburkholderia phytofirmans* PDOs	S-9
**Figure S9.** Structural comparison of the active sites of *Ac*PDO, *Mx*PDO and *Pp*PDO.	S-10
**Figure S10.** Structural comparison of cysteine residues between the *Ac*PDO, *Mx*PDO and the *Pp*PDO	S-11
**Figure S11.** Relative abundances of cysteine modifications in MALDI-TOF mass spectrometry fingerprinting	S-11
**Figure S12.** Differential scanning fluorimetry of wild type *Ac*PDO	S-12
**References**	S-12

Supplementary Information 2: File Ruhl_Supplement2_Alignments. Sequence alignments in FASTA format of the three subtypes of PDO based on the sequence set used by Xia *et al.* (2017)

Table S1: Oligonucleotides used in this study.

Primer name	Primer sequence (5'→3')[1]	Restriction enzyme	Purpose
*Acical*_ETHE1_fwd	aaaaatctagataacgagggcaaaaac-ATGTTATTCAAGCAGCTTTTTGACACCGA	*Xba*I	Ampification and ligation of the *pdo* gene with the pASK75 vector
*Acica*l_ETHE1_rev	GCTCTCGAGTTCATGTGGATTGCTCCCGA-TGTCGTC		
ask1b_fwd	AGAGTTATTTACCACTCCCTATCAG	-	Sequencing of vector constructs and mutants
ask2_rev	GCGTGGAGATCCGTGACGCA		
*Ac*PDO_C87A_fwd	gcTGCaGACCGCAAGGTGG	*Pst*I	Mutagenesis of cysteine codons in the *pdo* gene of *A. caldus*
*Ac*PDO_C87A_rev	ATCGGCTCCCGCGGC		
*Ac*PDO_C117A_fwd	gcCGTGAGCTATCGtTGGCAC	*Sma*I	
*Ac*PDO_C117A_rev	CCcGGgCGTGTGGCCGGGTGT		
*Ac*PDO_C117S_fwd	aGtGTGAGCTATCGCTGGCAC	*Bsa*JI	
*Ac*PDO_C117S_rev	cCCCGGCGTGTGGCCG		
*Ac*PDO_C137A_fwd	gcCGGcCGTACCGACTTTCAG	*Eag*I	
*Ac*PDO_C137A_rev	GCCGCCAATGAGCAGAGCGTC		
*Ac*PDO_C180A_fwd	gcgATCGCCGAAGAGAAACGCAGT	*Mbo*I	
*Ac*PDO_C180A_rev	ACTGACCCAGCGCCCGTGATA		
*Ac*PDO_C224A_fwd	gcCGGtCGtGAtGACATCGGGAGC	Δ*Not*I	
*Ac*PDO_C224A_rev	ACGCACATTAGCAGGGACCGCTACGTG		
*Ac*PDO_R139A_fwd	gcTACCGACTTTCAGGGCGGC	*Mwo*I	Mutagenesis of codons of puative substrate binding amino acid residues in the *pdo* gene of *A. caldus*
*Ac*PDO_R139A_rev	gCCGCAGCCGCCAATGAG		
*Ac*PDO_Y173A_fwd	gcTCACGGcCGCTGGG	*Eag*I	
*Ac*PDO_Y173A_rev	GTCGTGCCCGGGATAGACCAAG		
*Ac*PDO_P211A_fwd	AAACACATaCACGTAGCGGTCCCC	*Afl*III	
*Ac*PDO_P211A_rev	GGcTTGGGCCAGATCCAGG		
*Ac*PDO_K212A_fwd	gcACACATaCACGTAGCGGTCCC	*Afl*III	
*Ac*PDO_K212A_rev	GGGTTGGGCCAGATCCAGGG		
*Ac*PDO_T13A_fwd	gCCTACACCTACATCCTGGGCG	*Taq*I	Mutagenesis of codons of putative amino acids involved in the hydrogen bond network around the active site and the iron atome in the *pdo* gene of *A. caldus*
*Ac*PDO_T13A_rev	GCTGCTCTCGGTGTCgAAAAGCTG		
*Ac*PDO_T56A_fwd	gCCCACGTCCATGCGGACCAC	*Afl*III	
AcPDO_T56A_rev	TTCCAAcGCGTAGCGCAGGGTC		
*Ac*PDO_H59A_fwd	gcaGCtGACCACGTCAGCG	*Pvu*II	
*Ac*PDO_H59A_rev	GACGTGGGTTTCCAAAGCGTAGC		
*Ac*PDO_D61A_fwd	GcaCACGTCAGCGCTGC	*Afl*III	
*Ac*PDO_D61A_rev	CGCATGGACGTGGGTTTCC		
*Ac*PDO_H62A_fwd	gcCGTCAGCGCTGCC	*Rsa*I	
*Ac*PDO_H62A_rev	GTCCGCATGtACGTGGGTTTCC		
*Ac*PDO_T110A_fwd	gCaCCtGGCCACACGCCG	*Msc*I	
*Ac*PDO_T110A_rev	TGCCAGTACGCGGATGGC		
*Ac*PDO_T128A_fwd	gCcGGCGACGCTCTGCTCATTG	*Msp*I	
*Ac*PDO_T128A_rev	GAATACGCGATCGTGCCAGCGATAG		
*Ac*PDO_H171A_fwd	TATCACGGGCGCTGGGTCAG	*Nar*I	
*Ac*PDO_H171A_rev	GTCGgcgCCGGGATAGACC		
*Ac*PDO_H57A_fwd	gcCGTgCATGCGGACCACaTCAGCGCTG	*Sph*I	
*Ac*PDO_H57A_rev	cGTTTCCAAAGCGTAGCGCAGcGTCAG		
*Ac*PDO_H57G_fwd	ggCGTCCATGCGGACCAC	*Mwo*I	
*Ac*PDO_H57G_rev	GGTcTCCAAAGCGTAGCGCAGG		
*Ac*PDO_H113A_fwd	gcCACGCCGGGCTGCGTGAGCTATC	*Nar*I	
*Ac*PDO_H113A_rev	GCCGGGTGTTGCCAGTACGCGGATG		

[1]Restriction sites underlined; non-complementary nucleotides in lower case

Table S1 continued: Oligonucleotides used in this study.

*Ac*PDO_H113G_fwd	ggCACGCCGGGCTGC	*Mwo*I	
*Ac*PDO_H113G_rev	aCCGGGTGTTGCCAGTACGC		
*Ac*PDO_D130A_fwd	GcCGCTCTGCTCATTGGCGGCTGCGG	*Nar*I	Mutagenesis of codons of primary iron ligands in the *pdo* gene of *A. caldus*
*Ac*PDO_D130A_rev	GCCgGTGAATACGCGATCGTGCCAG		
*Ac*PDO_D130E_fwd	GAaGCTtTGCTCATTGGCGGCTGCGG	*Hind*III	
*Ac*PDO_D130E_rev	GCCgGTGAATACGCGATCGTGCCAG		
*Ac*PDO_D130H_fwd	cACGCTCTGCTCATTGGC	*Msc*I	
*Ac*PDO_D130H_rev	GCCaGTGAATACGCGAGC		

[1]Restriction sites underlined; non-complementary nucleotides in lower case

Table S2: Overview of specific activities of wild type *Acidithiobacillus caldus* PDO (*Ac*PDO) and its variants, iron content, numbers of preparations including total number of assays and T_m values.

	**Specific activity**	**Fe loading**	**Protein**	**# Preps | # Assays**	**Tm**	**# Preps | # Assays**
	U/mg	*mol/subunit*	*mg/L medium*		*°C*	
Wt	61.6 ± 3.5	0.77 ± 0.10	26.3	18 | 39	63.0 ± 1.3	5 | 15
T_{13}A	31.5 ± 3.9	0.80 ± 0.04	38.9	1 | 3	n.d.[2]	-
T_{56}A	-	-	-[1]	3 | 0	-	-
H_{59}A	4.1 ± 0.1	0.45 ± 0.02	25,1	1 | 3	n.d.	-
D_{61}A	≤ 0.05	0.53 ± 0.01	36.4	1 | 6	61.0 ± 0.05	1 | 3
H_{62}A	≤ 0.05	0.76 ± 0.05	38.2	1 | 6	63.3 ± 0.05	1 | 3
C_{87}A	1.2 ± 0.1	0.73 ± 0.05	39.2	2 | 6	63.3 ± 1.4	3 | 8
T_{110}A	34.9 ± 2.1	0.75 ± 0.03	25.5	1 | 3	n.d.	-
T_{128}A	18.5 ± 1.9	0.39 ± 0.05	10.8	1 | 3	n.d.	-
C_{117}A	6.3 ± 0.3	0.65 ± 0.06	15.6	3 | 9	n.d.	-
C_{117}S	41.8 ± 3.2	0.86 ± 0.07	34.2	1 | 3	n.d.	-
C_{137}A	33.5 ± 2.0	0.81 ± 0.05	31.2	2 | 6	n.d.	-
R_{139}A	0.3 ± 0.04	0.78 ± 0.02	33.5	4 | 9	60.7 ± 0.8	1 | 3
D_{141}A	1.5 ± 0.2	0.50 ± 0.01	34.9	1 | 3	55.7 ± 0.2	1 | 3
H_{171}A	≤ 0.05	0.74 ± 0.20	26.2	3 | 9	54.9 ± 0.7	1 | 3
Y_{173}A	6.6 ± 0.7	0.68 ± 0.02	26.2	3 | 6	n.d.	-
C_{180}A	54.6 ± 4.7	0.79 ± 0.01	28.5	1 | 3	n.d.	-
P_{211}A	23.6 ± 0.6	0.84 ± 0.04	34.4	3 | 6	n.d.	-
K_{212}A	5.8 ± 0.4	0.88 ± 0.04	38.5	3 | 6	n.d.	-
C_{224}A	0.7 ± 0.06	0.97 ± 0.05	32.1	2 | 6	63.3 ± 0.05	2 | 6

[1]No protein was obtained
[2]n.d., not determined

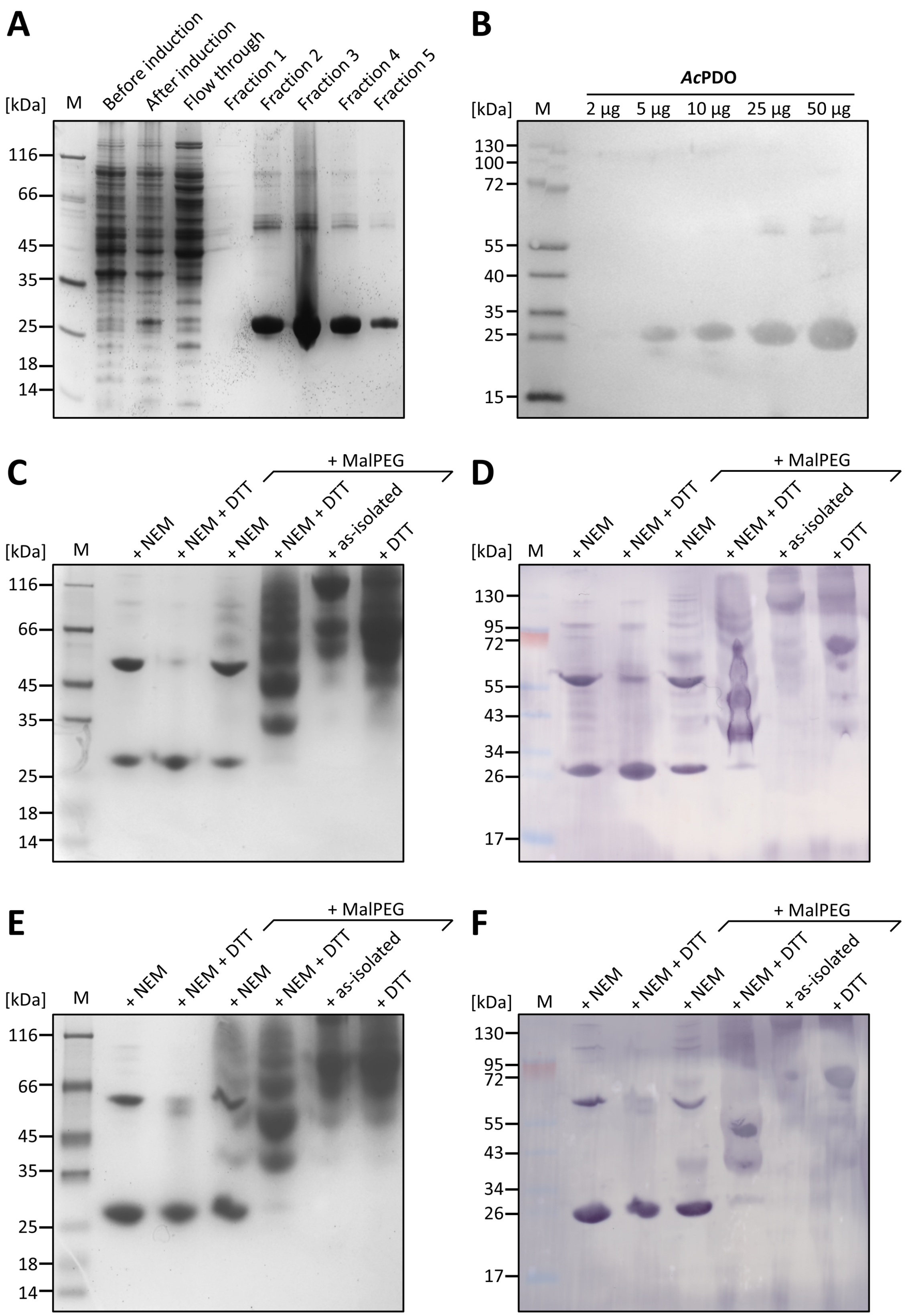

Figure S1. SDS Gel, MalPEG gel shift assays and Western analyses of the wild type and C_{87} and C_{224} variants of the *Acidithiobacillus caldus* PDO. **A**, 10% Tris-tricine polyacrylamide gel (Schägger and von Jagow, 1987) with total cell extracts before and after induction (from 100 µl of the culture); the flow-through of the Streptag column of the ultracentrifuge supernatant after cell disruption and the elution fractions 1-5 (10 µl each). **B**, Western analysis of increasing amounts of *Ac*PDO hybridized with horse-radish peroxidase-coupled Streptactin; M, marker proteins.; **C**, Coomassie-stained MalPEG gel shift assay of the *Ac*PDO C_{87}A variant and **D,** same with Western analysis using StrepMAP-Classic HRP-conjugated antibody (10-20 µg/lane). **E, F,** same as in panels C and D with the *Ac*PDO C_{224}A variant.

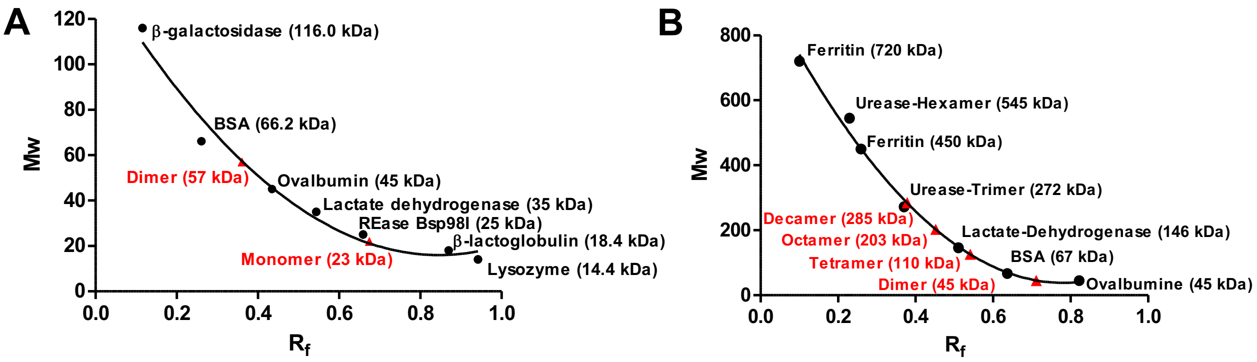

Figure S2. Calibration plot of R_f values of denaturing (**A**) and non-denaturing (**B**) polyacrylamide gel electrophoresis.

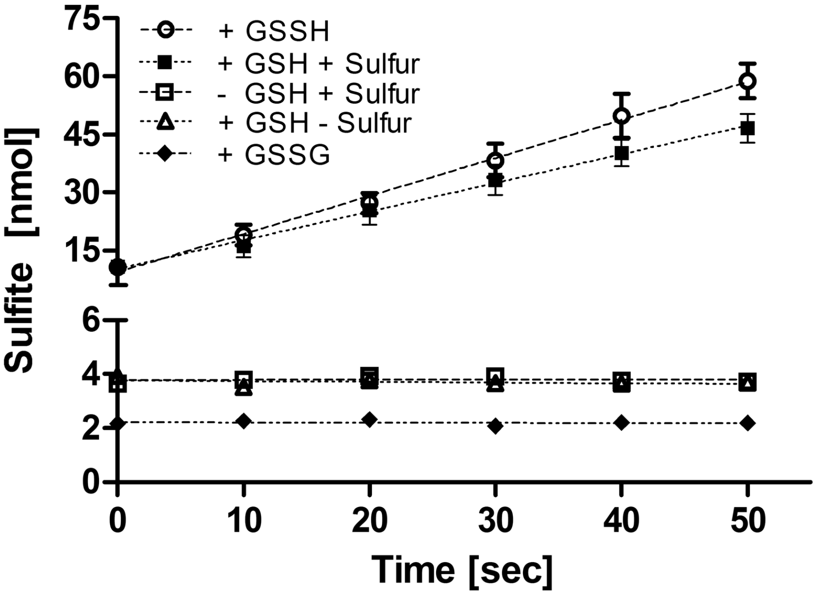

Figure S3. Activity profile of the *Ac*PDO. Sulfite formation of the wild type *Ac*PDO with 1 mM GSH, GSSH, or GSSG and/or 2 % elemental sulfur; 1 µg/ml of protein per assay and non-enzymatic controls.

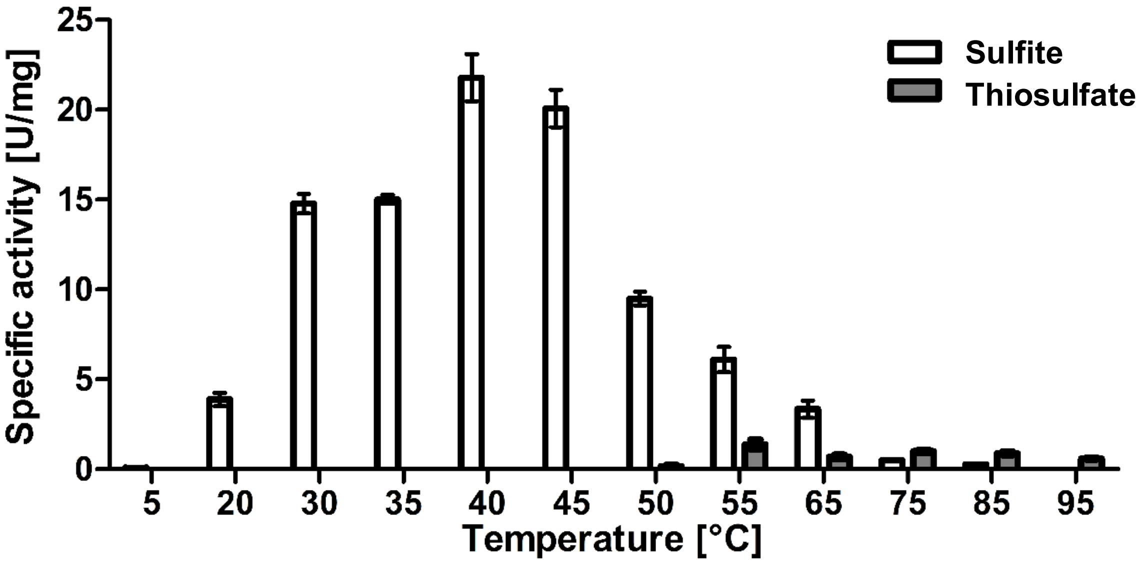

Figure S4. Formation of sulfite and thiosulfate during the enzyme reaction of the *Ac*PDO at pH 7.5 and different temperatures.

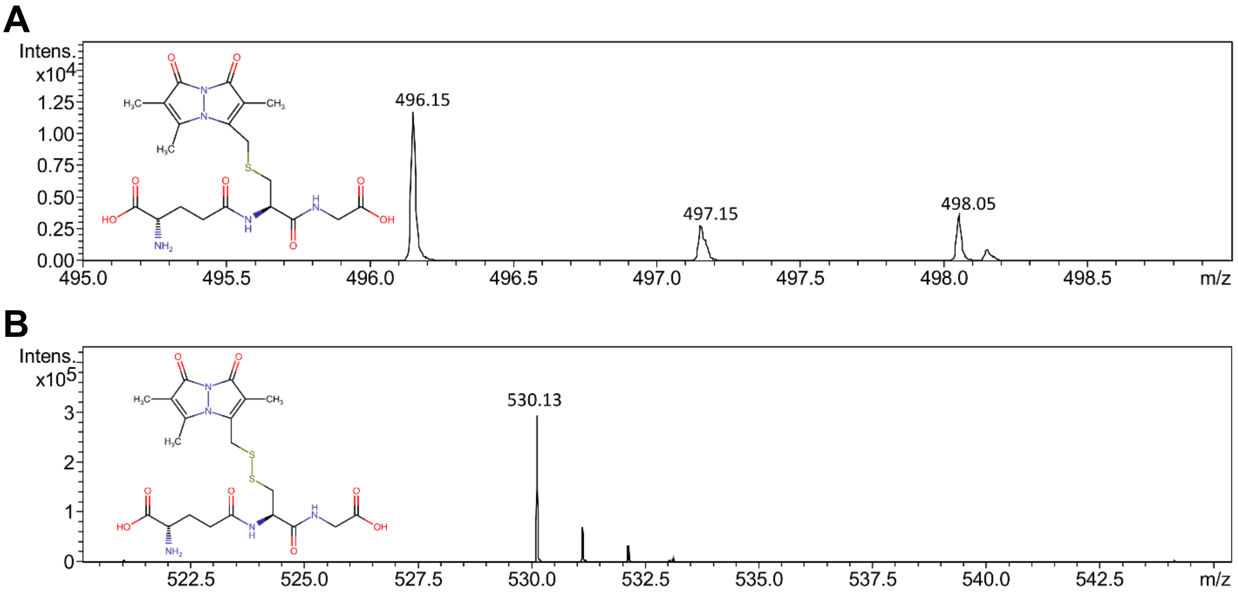

Figure S5. Mass spectra of GSH (**A**) and chemically synthesized GSSH (**B**) both derivatized with monobromobimane before analysis.

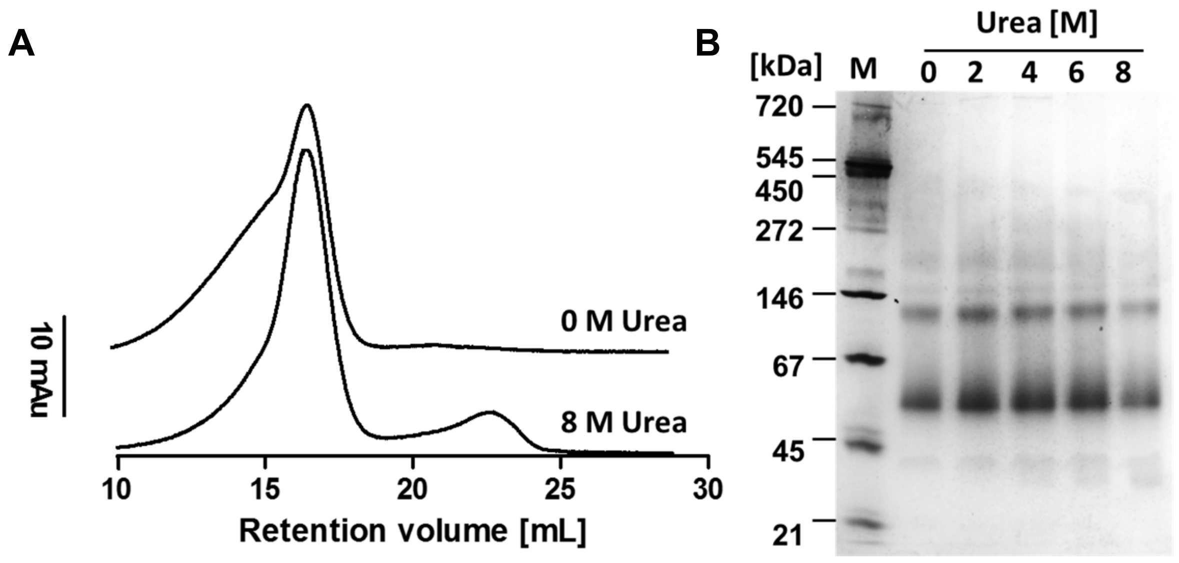

Figure S6. Urea denaturation experiments of wild type *Ac*PDO. **A,** Merged gel permeation chromatograms of *Ac*PDO before and after denaturation with 8 M urea. **B,** Native polyacrylamide gel after treatment of the *Ac*PDO with different concentrations of urea.

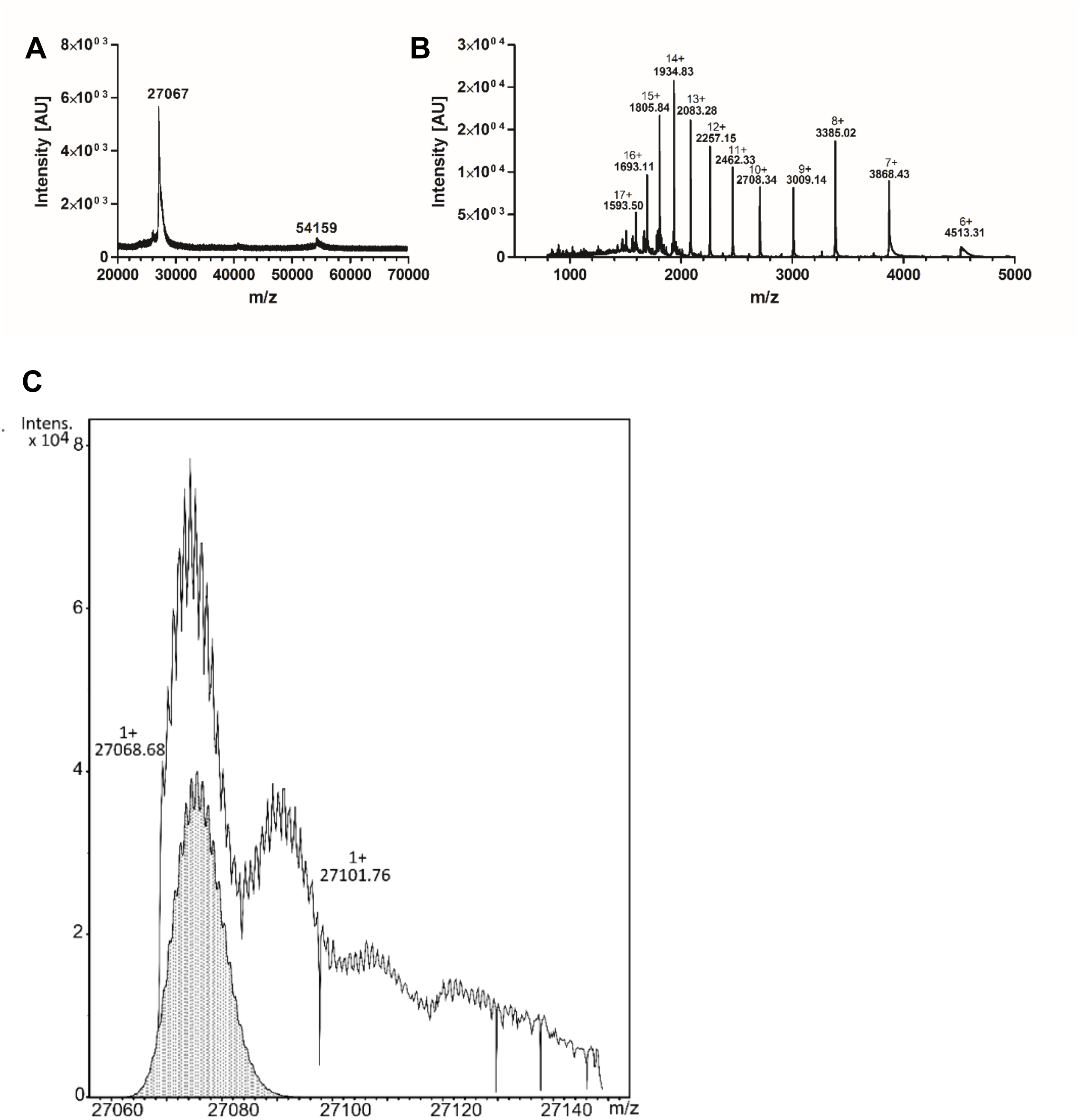

Figure S7. Mass spectra of the *Ac*PDO holoenzyme. **A,** MALDI-TOF spectrum. **B,** ESI spectrum (positive ionization mode). **C,** line, deconvoluted ESI spectrum; grey shaded area, calculated spectrum.

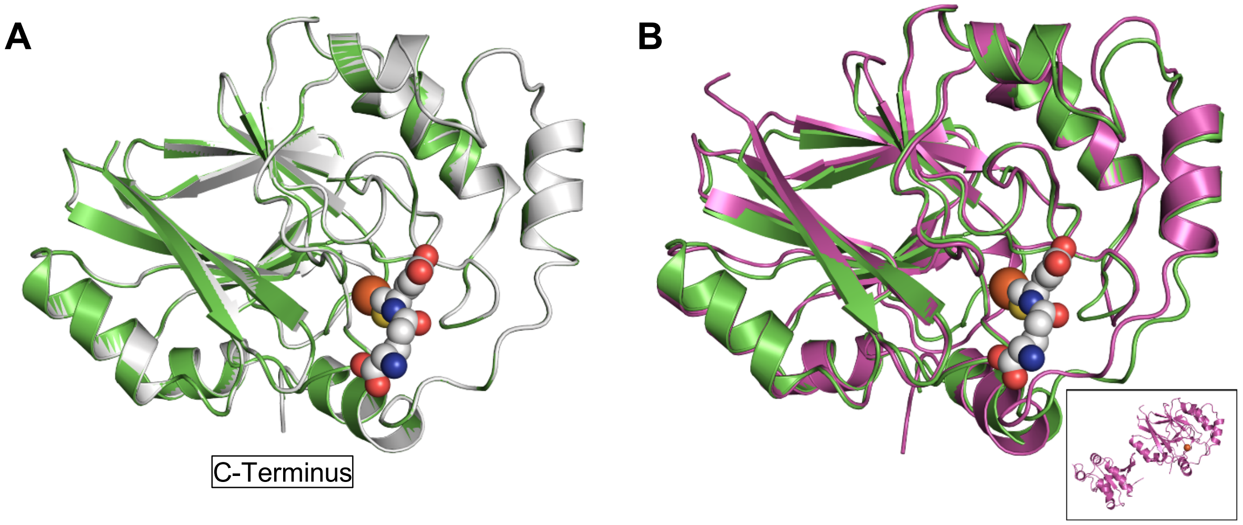

Figure S8. Comparison of the *Ac*PDO model with 3D structures of PDOs from *Myxococcus xanthus* (*Mx*PDO) and *Paraburkholderia phytofirmans* (*Pp*PDO). **A,** Comparison of the *Ac*PDO (green) with the *Mx*PDO (grey; PDB accession number: 4YSB) (Sattler et al., 2015). **B,** Comparison of the *Ac*PDO with the *Pp*PDO (purple; PDB accession number: 5VE5) (Motl et al., 2017); spheres, GSH and iron from *Pp*PDO structure; box, holoenzyme of the *Pp*PDO with C-terminally fused rhodanese domain.

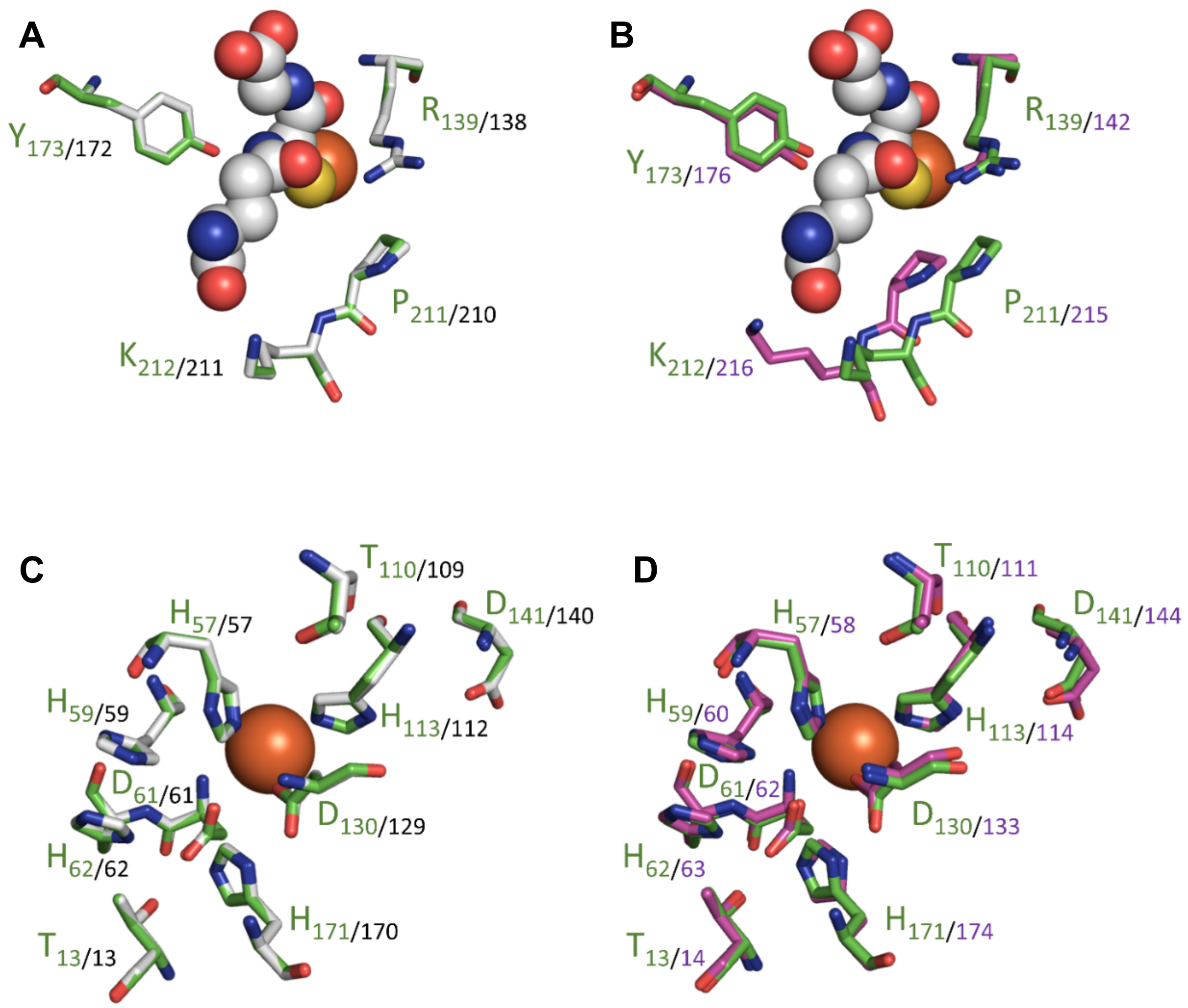

Figure S9. Structural comparison of the predicted active sites of the *Ac*PDO 3D model (green), *Mx*PDO (grey) and *Pp*PDO (purple) focussing on exchanges of amino acids putatively taking part in substrate binding (**A, B**) and in the hydrogen bonding network around the active site pocket (**C, D**).

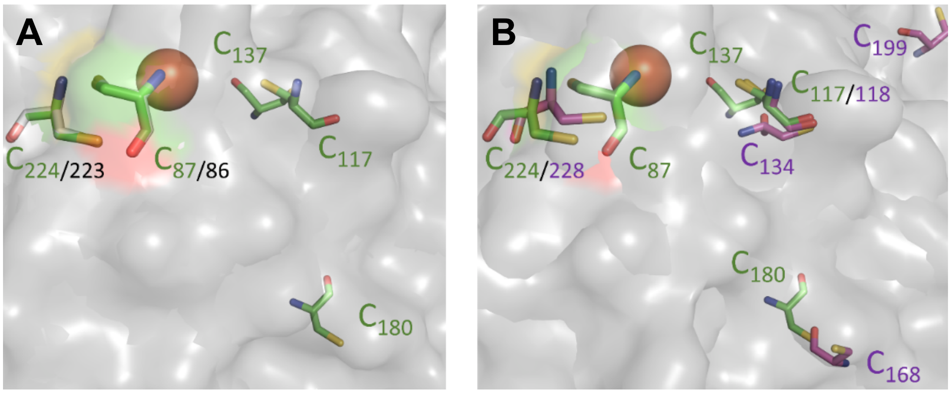

Figure S10. Structural comparison of cysteine residues between (**A**) the *Ac*PDO model (green) and *Mx*PDO (grey) and (**B**) the *Ac*PDO model (green) and the *Pp*PDO (purple).

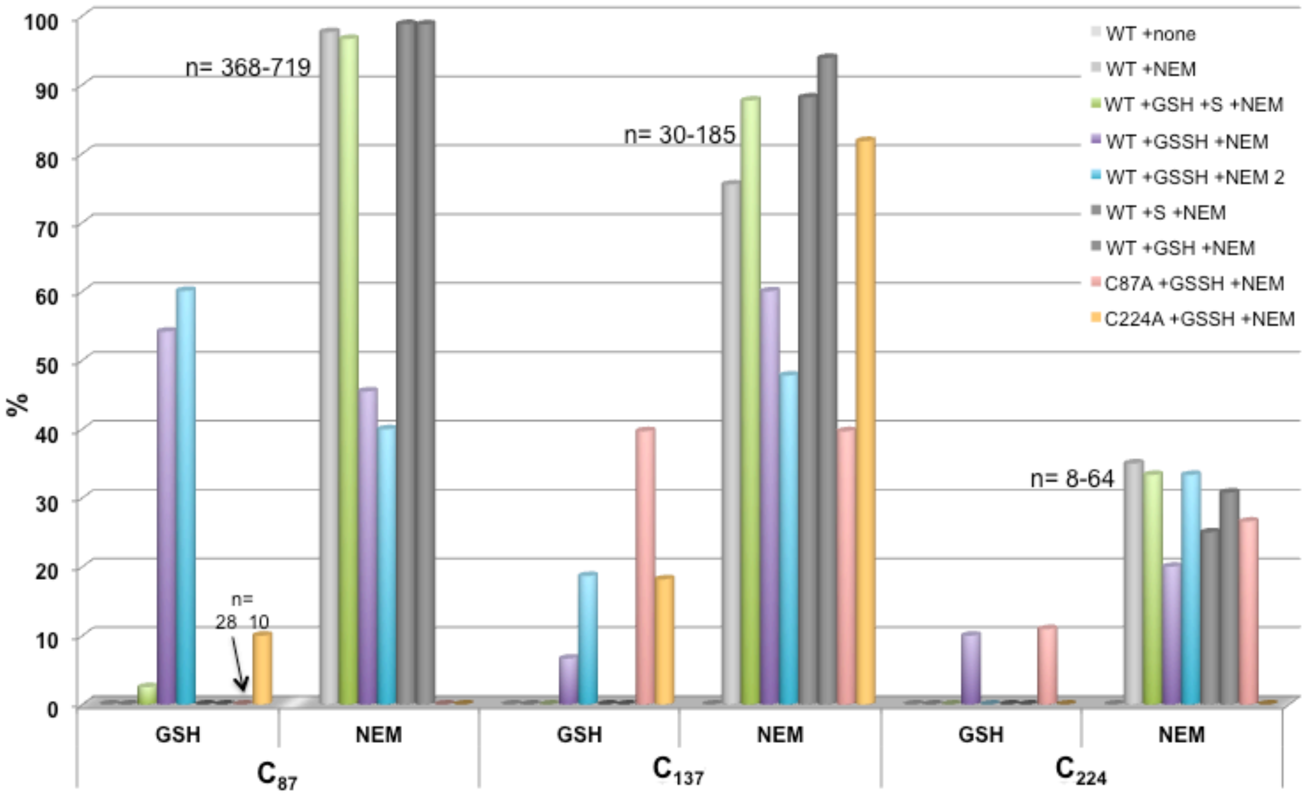

Figure S11. Relative abundances of cysteine modifications in MALDI-TOF mass spectrometry fingerprinting after treatment of the *Ac*PDO with various substrates for 1 min each in enzyme reaction buffer (70 mM Tris/HCl pH 7.5, 0.1% Tween20), followed by NEM derivatization; GSH and GSSH, each 1 mM; S, 2% sulfur flower; n represents the total number of fragment spectra recorded with cysteine modification; WT, wild type; C87A and C224A, cysteine variants.

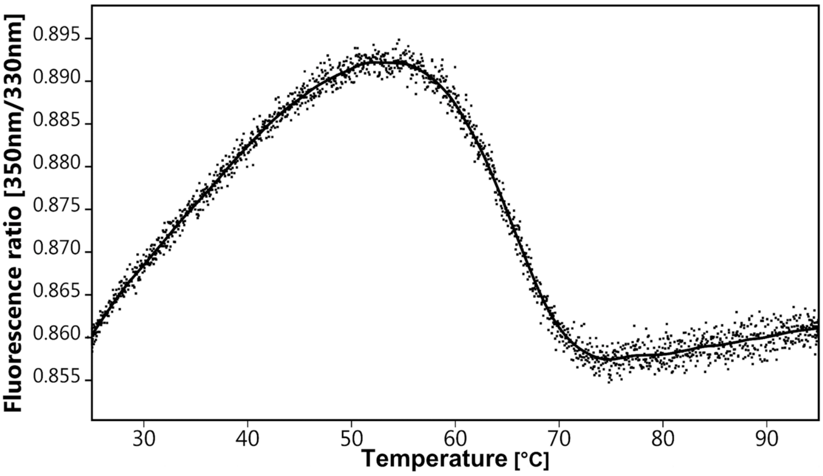

Figure S12. Differential scanning fluorimetry (tryptophane/tyrosine fluorescence) of wild type *Ac*PDO (1 mg/ml), measured in buffer E at a heating rate of 1 K / min.

References

Motl, N., Skiba, M.A., Kabil, O., Smith, J.L., and Banerjee, R. (2017). Structural and biochemical analyses indicate that a bacterial persulfide dioxygenase-rhodanese fusion protein functions in sulfur assimilation. *J Biol Chem* 292**,** 14026-14038. doi: 10.1074/jbc.M117.790170.

Sattler, S.A., Wang, X., Lewis, K.M., Dehan, P.J., Park, C.M., Xin, Y., Liu, H., Xian, M., Xun, L., and Kang, C. (2015). Characterizations of Two Bacterial Persulfide Dioxygenases of the Metallo-beta-lactamase Superfamily. *J Biol Chem* 290**,** 18914-18923. doi: 10.1074/jbc.M115.652537.

Schägger, H., and Von Jagow, G. (1987). Tricine-sodium dodecyl sulfate-polyacrylamide gel electrophoresis for the separation of proteins in the range from 1 to 100 kDa. *Anal. Biochem.* 166**,** 368-379.

Xia, Y., Lu, C., Hou, N., Xin, Y., Liu, J., Liu, H., and Xun, L. (2017). Sulfide production and oxidation by heterotrophic bacteria under aerobic conditions. *ISME J.* doi: 10.1038/ismej.2017.125.
